# Helical stability of the GnTV transmembrane domain impacts on SPPL3 dependent cleavage

**DOI:** 10.1038/s41598-022-24772-8

**Published:** 2022-12-05

**Authors:** Alkmini A. Papadopoulou, Walter Stelzer, Mara Silber, Christine Schlosser, Charlotte Spitz, Martina Haug-Kröper, Tobias Straub, Stephan A. Müller, Stefan F. Lichtenthaler, Claudia Muhle-Goll, Dieter Langosch, Regina Fluhrer

**Affiliations:** 1 Biochemistry and Molecular Biology, Institute of Theoretical Medicine, Faculty of Medicine, University of Augsburg, Universitätstrasse 2, 86159 Augsburg, Germany; 2 Lehrstuhl für Chemie der Biopolymere, Technische Universität München, Weihenstephaner Berg 3, 85354 Freising, Germany; 3 Institute for Biological Interfaces 4, Karlsruhe Institute of Technology, 76344 Eggenstein-Leopoldshafen, Germany; 4 Core Facility Bioinformatics, Biomedical Center, Ludwig Maximilians University Munich, 82152 Planegg-Martinsried, Germany; 5 DZNE – German Center for Neurodegenerative Diseases, Munich, Germany; 6 Neuroproteomics, School of Medicine, Klinikum rechts der Isar, Technical University of Munich, 81675 Munich, Germany; 7 Munich Cluster for Systems Neurology (SyNergy), Munich, Germany; 8 Institute of Organic Chemistry, Karlsruhe Institute of Technology, 76131 Karlsruhe, Germany

**Keywords:** Biochemistry, Biophysical chemistry, Proteases, Protein folding, Proteolysis

## Abstract

Signal-Peptide Peptidase Like-3 (SPPL3) is an intramembrane cleaving aspartyl protease that causes secretion of extracellular domains from type-II transmembrane proteins. Numerous Golgi-localized glycosidases and glucosyltransferases have been identified as physiological SPPL3 substrates. By SPPL3 dependent processing, glycan-transferring enzymes are deactivated inside the cell, as their active site-containing domain is cleaved and secreted. Thus, SPPL3 impacts on glycan patterns of many cellular and secreted proteins and can regulate protein glycosylation. However, the characteristics that make a substrate a favourable candidate for SPPL3-dependent cleavage remain unknown. To gain insights into substrate requirements, we investigated the function of a GxxxG motif located in the transmembrane domain of N-acetylglucosaminyltransferase V (GnTV), a well-known SPPL3 substrate. SPPL3-dependent secretion of the substrate’s ectodomain was affected by mutations disrupting the GxxxG motif. Using deuterium/hydrogen exchange and NMR spectroscopy, we studied the effect of these mutations on the helix flexibility of the GnTV transmembrane domain and observed that increased flexibility facilitates SPPL3-dependent shedding and vice versa. This study provides first insights into the characteristics of SPPL3 substrates, combining molecular biology, biochemistry, and biophysical techniques and its results will provide the basis for better understanding the characteristics of SPPL3 substrates with implications for the substrates of other intramembrane proteases.

## Introduction

Signal Peptide Peptidase-Like 3 (SPPL3) is an intramembrane aspartyl protease. The concept of proteolysis within the hydrophobic environment of cellular membranes has been well established and special characteristics mainly of the intramembrane proteases, but also of the substrates make it feasible^1–3^. In addition to SPPL3, four other members of the signal peptide peptidase/signal peptide peptidase-like (SPP/SPPL) family have been identified: SPP, SPPL2a, SPPL2b and SPPL2c^4–6^. Presenilin (PS) 1 and PS2, which comprise the presenilin family^7–11^, complete the representatives of aspartyl intramembrane proteases in humans. Apart from intramembrane aspartyl proteases, three additional classes of intramembrane cleaving enzymes have been identified: intramembrane metalloproteases represented by S2P, which is involved in cholesterol homeostasis and other intracellular signal transductions; intramembrane serine proteases that comprise the rhomboids and that have been first discovered in context of EGF-Receptor activation^12^; and, recently, Rce1 the first intramembrane glutamyl protease^13^.

Intramembrane aspartyl proteases share several common characteristics. They all have nine transmembrane (TM) domains and two aspartic acids at their active site^14,15^. The aspartic acids are located within conserved motifs, a YD motif in TM domain 6 and a GxGD motif in TM domain 7. Based on this, intramembrane aspartyl proteases are often also referred to as GxGD-proteases^16^. They also all comprise a conserved PAL motif in TM domain 9 that is predicted to be involved in the recognition of the substrate and to act as a gate keeper^17^. However, intramembrane aspartyl proteases also evince some differences. For instance, PSs require the formation of a tetrameric complex, termed γ-secretase, to be catalytically active, while SPP/SPPLs are catalytically active as monomers^18^. In addition, the members of these two families have an inverted membrane topology, with PSs having their N-terminus in the cytosol, while that of SPP/SPPLs localizes to the lumen/extracellular space. Consequently, their active sites are also inverted, and it is believed that this is the reason why PSs only target type-I TM proteins (N_out_), while SPP/SPPLs specifically recognize type-II TM proteins (N_in_)^14^.

Typically, aspartyl intramembrane proteases prefer substrates with rather short ectodomains that are either naturally short or have been shortened by a canonical sheddase^19^. However, SPPL3 was discovered as an exception, since it directly cleaves type-II TM proteins with long extracellular domains or type-II TM domains of multipass TM proteins^20,21^. In contrast, other members of the SPP/SPPL family only cleave selected substrates with long extracellular domains, most likely depending on the flexibility of the helix of the substrates TM domain^22^. Due to its independence on preceding shedding, SPPL3 has been termed a non-canonical sheddase, a term which so far had only been attributed to members of the rhomboid protease family^19^. Functional and proteomic analysis revealed SPPL3 to be a Golgi-localized protease that also in vivo cleaves numerous full-length, mature glycosidases and glycosyltransferases (GTs) in the Golgi, including N-acetylglucosaminyltransferase V (GnTV)^21,23^. SPPL3-mediated cleavage of GTs results in secretion of soluble GTs that contain the enzymes’ active site and thus, results in their deactivation within the cell, reducing glycosylation activity. A balance in SPPL3’s activity is crucial to maintain a balanced glycosylation pattern of the cell’s glycoproteins, as increased activity of SPPL3 would lead to hypoglycosylation of glycoproteins and vice versa^21^.

Very recently, non-canonical shedding of TNFα by SPPL2a in cultured cells was discovered^22^. Although SPPL2a preferentially cleaves TNFα after its ectodomain has been secreted by canonical sheddases, it is also capable of directly cleaving full-length TNFα^22^. Whether SPPL2a also acts as a non-canonical sheddase on other substrates remains unknown. In contrast, SPPL2b, the closest homologue of SPPL2a, does not accept full length TNFα as a *bona fide* substrate but requires an increased helix flexibility within the TM domain of TNFα to act as a non-canonical sheddase^22^. Whether non-canonical TNFα shedding also occurs in vivo and the physiological relevance of it remain unclear.

While some of the rhomboid proteases, which act as non-canonical sheddases, appear to have rather strict substrate requirements including also specific amino acid sequences around the actual cleavage site of the substrate^24^, no such observation has been made for any of the intramembrane aspartyl proteases. However, studies of PS1, SPP, SPPL2a and SPPL2b substrates identified some of their crucial requirements^17,22,25–28^. Since TM domains of substrate proteins that are targeted by intramembrane proteases are mainly α-helical in the unbound state, it is likely that they have to unfold when entering the interior of their cognate enzyme. High resolution structures for the γ-secretase complex with its substrates β-Amyloid-Precursor Protein (βAPP) and Notch, suggest that these substrates indeed unfold after their initial interaction with the enzyme^17,29^. In addition, a GG-hinge in the centre of the TM domain of βAPP, a well-studied γ-secretase substrate and key player in Alzheimer’s Disease pathology, allows bending motions thought to facilitate its endoproteolysis by PS1^30,31^. In line with that, processing of Bri2 by SPPL2b is promoted by a single helix-destabilizing glycine residue in the N-terminal half of the Bri2 TM domain^27^ and cleavage of TNFα at the C-terminal border of the TM domain by SPPL2a and SPPL2b is greatly facilitated by increased helix flexibility in the N-terminal part of the TNFα TM domain^22^. Thus, it is believed that the helical flexibility of the substrates TM domain has a major impact on its cleavability. Furthermore, many TM domains comprise GxxxG motifs that may cause their homodimerization^32^ and/or enhance their conformational flexibility^33^. Such motifs have been previously observed in the SPPL2a and SPPL2b substrate Bri2^27^ and in γ-secretase substrates, including βAPP^34,35^ and the p75 neurotrophin receptor^36^. Homodimerization of the substrate is not a requirement for γ-secretase cleavage, but nonetheless, mutations in the GxxxG motif affected cleavage efficiency of the p75 neurotrophin receptor, as well as of βAPP (Sykes et al.^36^; Munter et al.^34^; Eggert et al.^35^). However, the analysis of the GxxxG motif in Bri2 revealed that it is not crucial for cleavage by SPPL2b^27^.

As SPPL3 turns out to be one major regulator of cellular glycosylation profiles that play key roles, for instance in cancer development and metastasis^37^, it is of interest to understand what characteristics make Golgi-located type-II transmembrane proteins appropriate substrates for SPPL3 mediated cleavage. The best characterised substrate of SPPL3 so far is GnTV encoded by the gene *MGAT5*^21^. This glucosyltransferase is localised in the *trans* Golgi network, where it branches N-acetylglucosamine (GlcNAc) on glycoproteins to from complex N-glycans^38^. GnTV is a type II TM protein with a very long luminal and short cytosolic domain^21^. GnTV harbours a GxxxG motif in its TM domain, but no dimerization of GnTV through this motif has been observed so far. The SPPL3 cleavage sites in GnTV have been mapped and SPPL3 was shown to produce 5 distinct fragments, differing by one amino acid each. Cleavages sites are located at the predicted border of the TM and the extracellular domain^21^.

The aim of this study is to determine the impact of the GxxxG motif on helix flexibility of the GnTV TM domain and understand its role in SPPL3 mediated cleavage. This reveals important information regarding the substrate requirements of the non-canonical sheddase SPPL3 that will help to answer the question whether GxxxG motifs in SPP/SPPL substrates have different impact on their cleavage than in γ-secretase substrates. By replacing the glycines of the GxxxG motif with different amino acids, the helical stability of the GnTV TM domain was affected. Reduction of the helical flexibility by introducing leucine residues reduced SPPL3 mediated cleavage of GnTV, while increased helical flexibility due to proline substitutions seemed to enhance cleavage.

## Results

### Mutations in the GxxxG motif of GnTV affect its cleavage

The presence of glycines in the TM domain of a protein is considered to augment the flexibility of the α-helix, as originally shown by circular dichroism spectroscopy and more recently by recording amide deuterium/hydrogen exchange (DHX) kinetics of various model TM domains^39,40^. Molecular dynamics simulations have revealed the mechanism behind this destabilizing effect. Specifically, a packing defect at glycine enhances local hydration and alters H-bond occupancies. These effects facilitate local TM helix bending at the glycine site and change the collective dynamics of the helix^40^. For this reason, the existence of two glycines in the TM domain of GnTV, as part of the GxxxG motif, is of particular interest and specific mutants were designed, aiming to either stabilise the presumably α-helical TM domain of GnTV, or to destabilise it even further.

**Figure 1:**
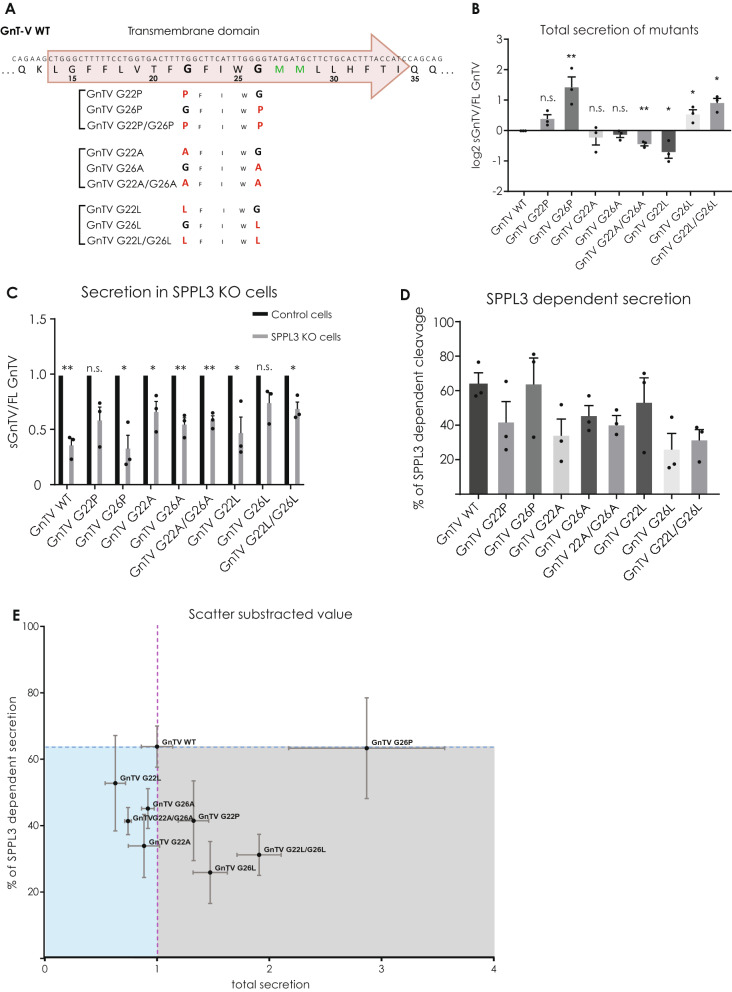
(**A**) Amino acids of the GnTV TM domain and its flanking regions are depicted using the single letter code together with the respective nucleotide triplets of the DNA coding region. The predicted TMD is annotated within the red arrow, which also depicts the N- to C-terminal orientation of the protein. The potentially α-helix destabilizing glycines are marked in bold. Below the sequence of GnTV the respective amino acid sequences of the three sets of mutations are shown. The mutated amino acids are marked in red. (**B**) Intracellular and secreted GnTV levels of the same sample were analysed. As lysates were analysed on different Western Blots than the corresponding supernatants, GnTV WT was used as a control and levels of sGnTV were always normalised to GnTV WT. To compare the turnover of all GnTV mutants, the log_2_ of soluble GnTV (sGnTV) divided by the full length GnTV (FL GnTV) is depicted in a linear scale. GnTV FL comprises the sum of GnTV_im_ and GnTV_mat_. To confirm the reproducibility of the experiments, three (n = 3) biological replicates were seeded and analysed on different weeks for each of the mutants, each biological replicate comprised three technical replicates. The values are quantified from Western blot analysis (Supplementary Fig. 1) and are normalized to the secretion of GnTV WT (0). Mean + SEM, multiple unpaired, two-tailed t tests with Holm-Sidak multiple comparisons correction. (**C**) For each mutant secretion of sGnTV by both SPPL3 and unknown protease(s) in HEK293 cells (Control) was set to 100% and the secretion of GnTV of the same mutant by only the unknown protease(s) in SPPL3 KO cells is depicted. n = 3 biological replicates with 3 technical replicates each, mean + SEM, two-tailed t test for each pair separately (**D**) Using the results of 1C, SPPL3 dependent secretion was calculated by subtracting percentage of secretion by the unknown protease from the secretion by both proteases. (**E**) On the x axis, the total secretion of each mutant compared to the WT is plotted. The results from Fig. 1B are being used, but instead of the log2 results the linear numbers with secretion of GnTV WT normalised to 1 were used. The y axis depicts the SPPL3 dependent secretion in percentage. Results from Fig. 1D where used. The values of GnTV WT are marked with the dotted lines. n.s. = non significant, *p < 0.05, **p < 0.01.

To this end, either one or both glycines of the GxxxG sequence were mutated (Fig. 1A) using amino acids expected to impact on the properties of the helix in different ways. Mutating glycine to proline (P) is expected to locally distort the α-helix, as its amide group lacks the hydrogen atom necessary to form a hydrogen bond with the main chain carbonyl oxygen at the (i-4) position along the helix and its bulky side chain ring structure clashes sterically with its (i-1) neighbour^41^. Mutations to alanine (A) are expected to provide more stability to the α-helix than glycines and finally, mutations to leucine (L) should offer the highest stability to the helical structure, since leucine can establish non-polar interactions between its large and flexible side chains along the helix^42^.

To analyse the turnover of wildtype GnTV (GnTV WT) and mutant proteins, T-Rex 293 (HEK293) cells, which express SPPL3 endogenously, were used. The GnTV protein transiently expressed in the HEK293 cells carries an N-terminal Flag tag and a C-terminal V5 tag, that do not disturb the maturation of the protein, as previously demonstrated^21^. Full length GnTV localizes to the Golgi and is detected as an immature (GnTV_im_) and a mature (GnTV_mat_) form^21^. GnTV_mat_ has a higher molecular weight due to its complex glycosylation and is the direct substrate of SPPL3. Cleavage of GnTV_mat_ by SPPL3 releases a soluble GnTV peptide (sGnTV) in the extracellular space and an intracellular peptide, which eludes detection due to rapid degradation^21^.

Single proline mutants (GnTV G22P and G26P) underwent normal maturation as the ratio between GnTV_im_ and GnTV_mat_ appears similar compared to GnTV WT (Supplementary Fig. 1A). On the contrary, the double proline mutant (GnTV G22P/G26P) completely lacked the band representing GnTV_mat_. Most likely, the strong interference of the two prolines with the formation of the GnTV G22P/G26P TM helix resulted in misfolding of the protein and, thus, failing ER-quality control mechanisms. Since GnTV G22P/G26P_im_ is less abundant compared to GnTV WT_im_ it is likely that this mutant is degraded in the early secretory pathway, but due to overexpression some misfolded protein still accumulates. In agreement with this, no sGnTV G22P/G26P was detected in the conditioned media (Supplementary Fig. 1A). Compared to GnTV WT, secretion of the GnTV G22P mutant tended to be slightly increased while secretion of GnTV G26P mutant was significantly increased (Fig. 1B).

The alanine mutations were expected to stabilise the α-helical structure of the GnTV TM domain only mildly resulting in a minimal effect on secretion, and indeed, all mutants matured and were cleaved properly, with the single mutations having a non-significant tendency to decrease the secretion of sGnTV (Fig. 1B and Supplementary Fig. 1B). The double mutant, GnTV G22A/G26A, showed a significant decrease in the secretion of sGnTV.

Surprisingly, the leucine mutants that were expected to have the strongest effect in stabilising the TM-helix, showed variable results. While, as expected, the GnTV G22L mutation, evinced a significant decrease in the secretion of sGnTV (Fig. 1B and Supplementary Fig. 1C), mutating the second glycine (GnTV G26L) or both glycines simultaneously (GnTV G22L/G26L), resulted in a significantly increased secretion (Fig. 1B and Supplementary Fig. 1C).

### SPPL3 cleaves GnTV mutants with different efficiencies

For some glycosyltransferases, other proteases than SPPL3 were shown to contribute to their secretion^43–45^ and also SPPL3 is most likely not the only protease responsible for cleavage and secretion of GnTV^21^. However, the additional proteases involved in secretion of GnTV remain elusive. To assess the SPPL3-specific effect of the GnTV mutations the CRISPR/Cas9 technique was employed to knock out SPPL3 from HEK293 cells (SPPL3 KO). A knockout single cell clone that had one base pair inserted in its genomic DNA resulting in an early stop codon was chosen (Supplementary Fig. 2). Determination of SPPL3-dependent cleavage of GnTV WT and each mutant in these cells relative to cells with endogenous SPPL3 expression provides an indirect way to assess SPPL3-dependent cleavage, since secretion of sGnTV in SPPL3 KO cells can be solely attributed to non-SPPL3 protease(s).

For GnTV WT, lack of SPPL3 significantly decreased secretion of sGnTV by about 60% (Fig. 1C). This goes in line with results observed earlier upon transient knock down of SPPL3^21^ and indicates that 60% of the GnTV WT shedding can be attributed to SPPL3 and the remaining 40% to the unknown protease(s). While GnTV G26P and G22L are cleaved by SPPL3 with similar efficiency as GnTV WT, all other mutations reduced SPPL3-dependent cleavage (Fig. 1C). To improve the presentation of these results, the percentage of SPPL3-dependent secretion was calculated. This reveals that SPPL3 has the tendency of cleaving GnTV G22P, G26L, the double mutant G22L/G26L and all the G to A mutants less efficiently compared to GnTV WT, with GnTV G26L and G22/G26L having the strongest effect (Fig. 1D). However, this only provides information regarding the contribution of SPPL3 to the secretion of each mutant, without considering the effect of the mutation on total secretion. This particularly distorts the information on the mutants which depict a significant change in total secretion, (Fig. 1B) but no change in the cleavage ratio between SPPL3 and the unknown protease(s), like GnTV G26P (Fig. 1C,D).

To consider both these aspects in one graph the total secretion of each mutant compared to the WT is plotted on the x-axis and SPPL3 dependent secretion in percentage is depicted on the y-axis (Fig. 1E). All mutants to the right of the purple line are in total secreted more than GnTV WT and all samples below the blue line are cleaved less by SPPL3. Conclusively, mutants within the grey area are more secreted, but not by SPPL3. The mutants in the blue area are less secreted and SPPL3 has also a lower preference for them, pointing to unfavourable mutations. GnTV G26P is the only mutant that is cleaved with unchanged preference from both proteases, but is also more secreted than GnTV WT. This indicates that GnTV G26P cleavage by SPPL3 and the unknown protease(s) can happen faster than cleavage of all other mutants analysed, probably due to a favourable conformation.

### Subcellular localisation and cleavage sites of selected mutants remain unchanged

To correlate cleavage efficiencies of the mutant GnTV proteins with potential changes in helical dynamics and structural changes three representative mutants were selected for further analysis. GnTV G26P represents the only mutant that is cleaved more efficiently by both SPPL3 and the unknown protease (Fig. 1E). GnTV G22L is cleaved less by SPPL3 and the unknown protease together, and by SPPL3 alone, as compared to GnTV WT (Fig. 1D,E). Finally, GnTV G22L/G26L is in total more efficiently secreted, but SPPL3 depicts a strongly reduced cleavage preference (Fig. 1B,D).

**Figure 2:**
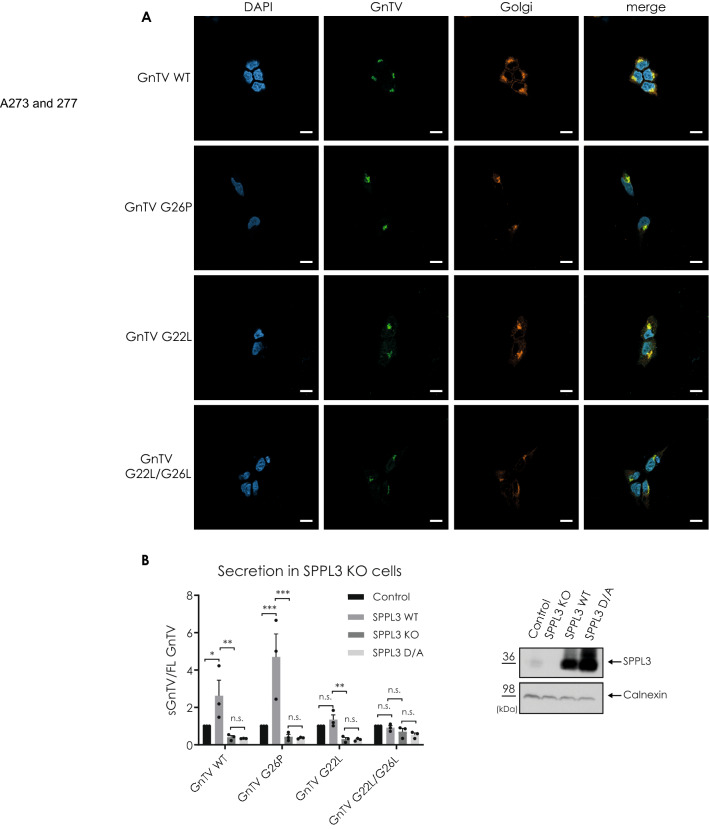
(**A**) HEK293 cells were stably transfected with N-terminally Flag and C-terminally V5-tagged GnTV WT, GnTV G26P, GnTV G22L or GnTV G22L/G26L and were co-stained with V5 to visualize GnTV and a trans-Golgi marker (TGN46). DAPI was used to stain the nuclei. White bar = 10 μm. (**B**) SPPL3 KO cells were stably transfected with either SPPL3 WT or catalytically inactive SPPL3 D272A (D/A). Expression levels are shown in Western Blot on the right (uncropped blots in supplements). Note that expression of the catalytically inactive variant is stronger than that of SPPL3 WT. The indicated GnTV variants were transiently expressed and secretion of GnTV (sGnTV) was quantified relative to intracellular GnTV (FL GnTV). Secretion was normalized to the secretion of the respective GnTV mutants in cells with endogenous expression of SPPL3 (Control). n = 3 biological replicates; mean + SEM, two-tailed t test for each pair separately. n.s. = non significant, *p < 0.05, **p < 0.01, ***p < 0.001.

SPPL3 and GnTV both localise to the Golgi, where the intramembrane cleavage occurs^21^. Mutations in the primary structure of the GnTV protein could cause mislocalisation in the secretory pathway and decrease shedding by SPPL3 due to differential localisation of protease and substrate. To exclude this, immunofluorescence verifies the subcellular localisation of the GnTV WT and mutants (Fig. 2A). As expected, GnTV WT was detected predominantly in the trans-Golgi as were all the selected GnTV mutant proteins. Thus, we conclude that the mutations in the GxxxG-motif selected for further analysis do not affect the localisation of the GnTV protein. This suggests that SPPL3 can interact with any of these mutants with a similar likelihood, as with GnTV WT. To further exclude that the effects on GnTV cleavage observed in SPPL3 KO cells are due to CRISPR/Cas9 off target effects or are indirectly mediated by non-catalytic functions of SPPL3, we stably expressed either SPPL3 WT or a catalytically inactive SPPL3 D272A variant (SPPL3 D/A) in SPPL3 KO cells and co-expressed GnTV WT or the selected mutants (Fig. 2B). Secretion of the individual sGnTV variants was normalized to the secretion of the respective mutant in cells with endogenous SPPL3 expression (Control). Secretion of all GnTV variants in cells overexpressing the catalytically inactive SPPL3 D/A was similar to that observed in SPPL3 KO cells (Fig. 2B). Demonstrating that effects on GnTV secretion depend on the catalytic activity of SPPL3 and corroborating that the residual shedding mediated by unknown protease(s) is independent of SPPL3 expression and activity. As expected, overexpression of SPPL3 WT rescued the knockout effect and further increased secretion of GnTV WT, while secretion of GnTV G26P was even further increased (Fig. 2B). In contrast co-expression of SPPL3 WT and GnTV G22L rescued the SPPL3 KO induced reduction of GnTV secretion but did not significantly increase secretion compared to endogenous SPPL3 expression. As observed before (Fig. 1C,D) GnTV G22L/G26L was predominantly cleaved by the unknown protease(s) and, consequently, SPPL3 KO reduced its secretion only mildly. Nonetheless, co-expression of SPPL3 rescued the knockout effect but did not increase secretion further. This again suggests that the leucine substitutions resulted in less favorable SPPL3 substrates.

**Figure 3:**
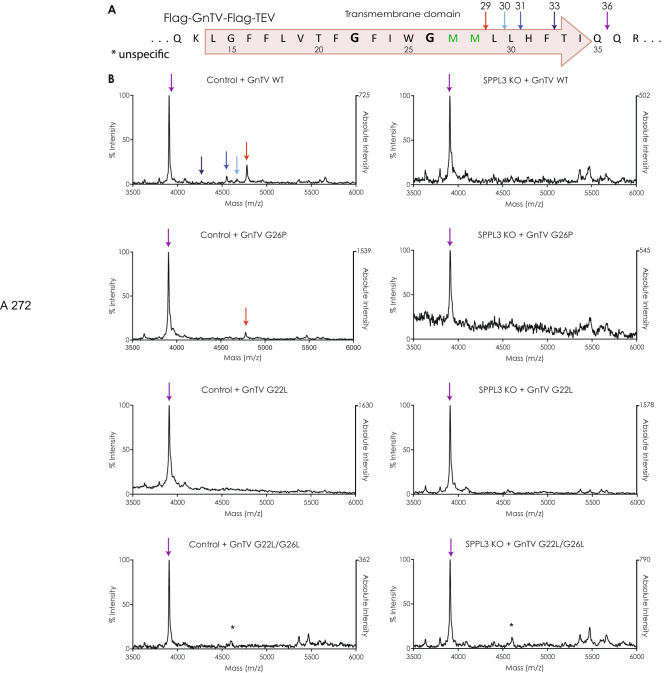
(**A**) Amino acid sequences of the TM domain of the GnTV peptide used in the mass spectrometric analysis. Small arrows indicate the peptides detected. (**B**) C-terminal cleavage sites of GnTV. Mass spectrometric analysis of fragments generated in presence (Control) or absence (SPPL3 KO) of SPPL3, respectively. Numbers indicate the position of the most C-terminal amino acid of the respective cleavage product. *marks unspecific peak. The intensities were normalized with the highest peak set at 100%. On the right side of each graph, the absolute intensity of the highest peak is indicated.

To assess whether these mutations affect the cleavage sites of SPPL3 in GnTV, mass spectrometry was used to determine the cleavage sites for each of the selected mutants. To allow detection of the C-terminal cleavage product by mass spectrometry, a TEV-cleavage site was inserted into the extracellular domain of GnTV to reduce the size of the secreted ectodomain so it can be detected by mass spectrometry (Supplementary Fig. 3). The analysis was performed in control HEK293 cells and SPPL3 KO cells stably expressing the respective GnTV mutants (Fig. 3). Analysis of GnTV WT in HEK293 cells with endogenous SPPL3 expression confirmed four of the five cleavage sites that have been previously identified^21^. The peptide bond between M28 and L29 was confirmed as the major cleavage site, with minor cleavage sites at positions L30, H31 and T33 (Fig. 3A,B). Interestingly, an additional cleavage site was detected at position Q36 C-terminally of the annotated GnTV TM domain. However, cleavage at this site was also present in SPPL3 KO cells, while all other cleavage sites were not detectable. This proves that the cleavage product starting from Q36 is SPPL3-independent, suggesting that cleavage at this site is mediated by the unknown protease(s). Since peptides starting at Q36 are shorter and less hydrophobic than peptides generated from SPPL3 cleavage, we assume that detection of Q36-peptides by mass spectrometry is much more efficient.

Analysis of GnTV G26P revealed cleavage at the major SPPL3 cleavage site. Since the cleavage product of the unrelated protease was also more prominent in this sample, detection of the more hydrophobic SPPL3 cleavage products may be even less effective. SPPL3 cleavage of GnTV G22L, which is in total significantly less secreted than GnTV WT, was hardly detected by mass spectrometry, most likely because the total amount of cleavage product was below the detection level. Similarly, no SPPL3 cleavage product was seen for GnTV G22L/G26L. In line with the results of the secretion analysis, this supports the hypothesis that SPPL3 cleaves GnTV with leucine substitutions in the GxxxG motif less efficiently. Moreover, the result on GnTV G26P suggests that the major cleavage site of SPPL3 in the mutant GnTV proteins remain the same as compared to GnTV WT and the cleavage site of the unknown protease(s) locates outside of the annotated TM domain.

### Mutations impact on the conformational flexibility of the GnTV TM helix

To relate the cleavability of the GnTV TM helix by SPPL3 to its conformational flexibility, we compared GnTV WT and the selected mutants by Deuterium/Hydrogen exchange (DHX), whose kinetics depend on the stability of amide H-bonds^46^. The analyses were performed using synthetic peptides corresponding to the predicted GnTV WT TM domain as well as the G26P, G22L, and G22L/G26L mutants (Table S1). Similar to previous analyses of various substrate TM domain peptides^30,47,48^, DHX kinetics of exhaustively (> 96%) deuterated peptides were measured in 80% trifluoroethanol (TFE), a solvent that roughly matches the polarity within the solvated interior of a protein^49^, such as an intramembrane protease^50^. To ensure proper α-helical folding, the secondary structure of the synthetic peptides was determined by circular dichroism (CD) spectroscopy in the same solvent. All spectra exhibit a line shape typical of α-helices and revealed a helix content of ~ 80% with minor variations (Supplementary Fig. 4).

**Figure 4:**
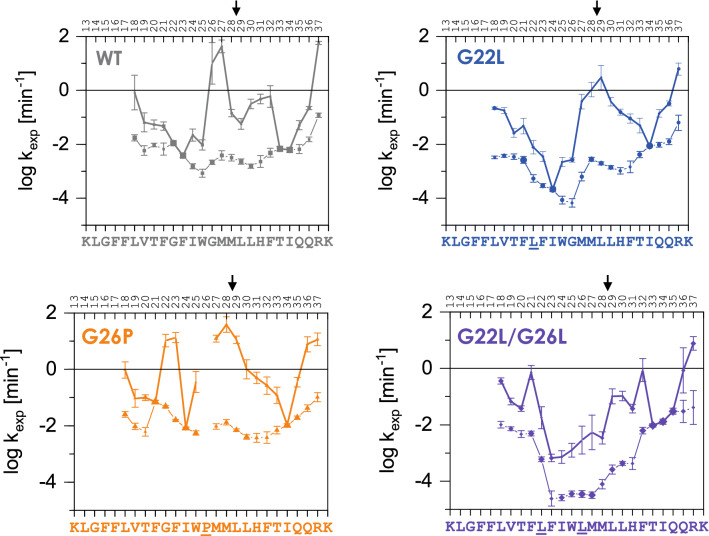
Conformational flexibility of GnTV TM helices probed by DHX. DHX rate constants of individual amides. Within regions of biphasic DHX, more positive values denote k_exp,A_ and the sizes of the data points approximate the deuteron populations A and B. Values characterizing single H-bond openings (k_exp_, k_exp,A_) are connected by thick lines. Error bars correspond to the errors of fit in k_exp_ determination from n = 3 independent DHX reactions. The main cleavage site is indicated by an arrow.

In the DHX experiments, gas-phase fragmentation by electron transfer dissociation (ETD)^47^ after different incubation periods yielded the residue-specific DHX kinetics (Supplementary Fig. 5). From the DHX kinetics, we derived the corresponding amide exchange rate constants k_exp_ (Fig. 4). Interestingly, a biexponential decay function proved to be more appropriate to fit most residue specific DHX kinetics of the GnTV TM domain peptides than the conventional monoexponential (Supplementary Figs. 5, 6 and 7). Amides where DHX is biexponential, or biphasic, are characterized by a quicker exchanging population A, yielding a high k_exp,A_, and a slower exchanging population B associated with a low k_exp,B_ (Fig. 4). We propose that k_exp,A_ and k_exp,B_ values describe DHX after H-bond opening at a given amide (‘single opening’) or of the more rarely occurring simultaneous opening of two neighbouring H-bonds (‘double opening), respectively (Supplementary Figs. 6 and 7). Thus, biphasic DHX kinetics appear to diagnose very flexible parts within a helix showing double openings in addition to single ones (as detailed in the Supplementary Discussion).

In GnTV WT, monoexponential DHX is only seen at G_22_F_23_ and at T_33_I_34_ (Fig. 4 and Supplementary Fig. 5). In contrast, the region in between exhibits pronounced biphasic exchange kinetics, as well as rather high k_exp,A_ values, especially at G_26_M_27_. Therefore, these H-bonds are of only marginal stability. Biphasic DHX upstream of G22 and downstream of I34 suggests double H-bond openings resulting from the deformations associated with helix fraying, a general phenomenon seen at helix termini^51^. Together, the biphasic nature of the amide exchange and high k_exp_ values within the central region attest to an exceptional conformational flexibility of the GnTV WT TM helix. The flexibility of its internal part is most pronounced at sites directly downstream of G22 and G26, in accordance with the known helix-destabilizing effect of glycine^39,40^. DHX profiles of the GnTV TM domain mutants strongly deviate from those of GnTV WT at many internal positions, while being comparable near their termini (Fig. 4). Specifically, the G26P mutation promotes both single and double H-bond openings at upstream and downstream amides. By contrast, the G22L mutation stabilizes the helix downstream of position 22. The G22L/G26L mutant has an even more pronounced stabilizing impact extending further towards the N-terminus. Apart from decreasing k_exp_,_A_, the mutations to Leu also attenuated the biphasic nature of DHX between G22 and G26 (G22L mutant) and between G22 and L29 (G22L/G26L mutant), respectively.

### Mutations destabilize and lead to changes in helix curvature

**Figure 5:**
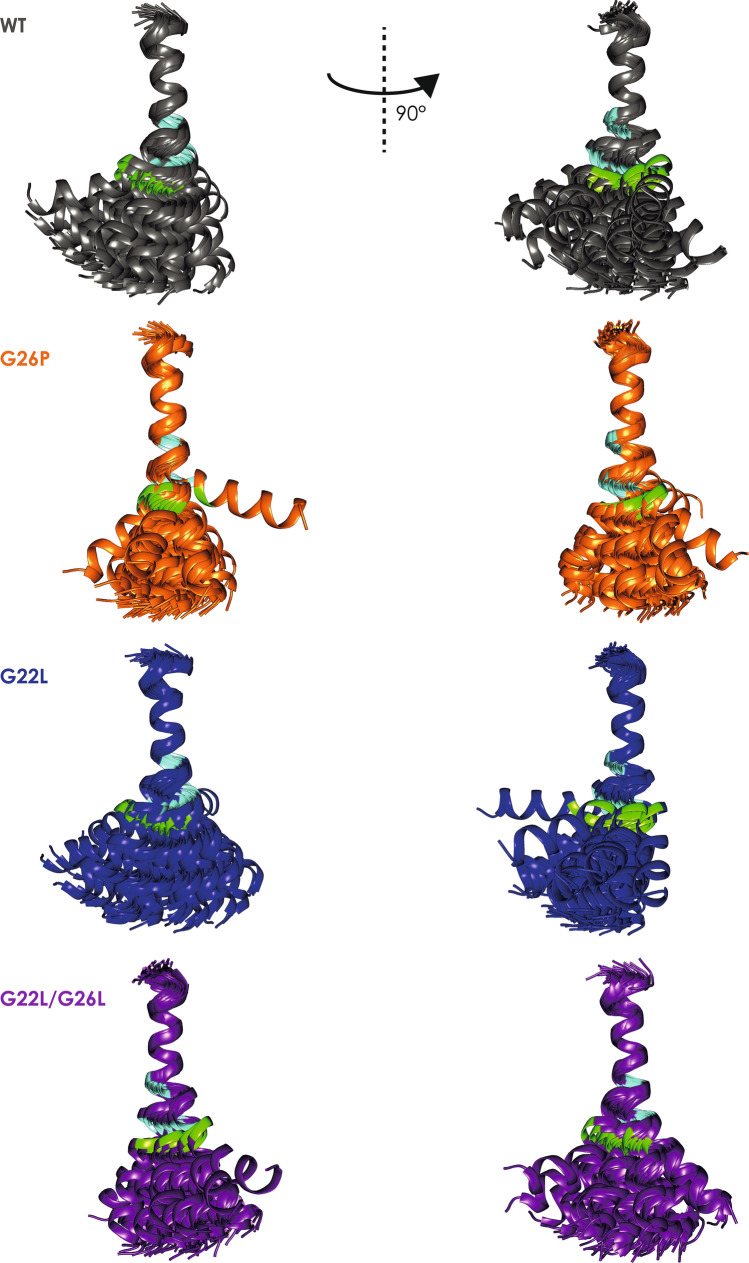
Structural bundles aligned on residues G15–G/L22. The 50 lowest energy structures out of 200 are shown. Green: M28, indicating the major SPPL3 cleavage site; light blue G/L22 and G/L/P26.

As previous studies on C99-TM domain^52^ and TNFα-TM domain^22^ mutants demonstrated that mutations in the TM domain can impact on its structure and stability in a way that is not easily predictable, we determined the structures of the GnTV WT and the selected mutant TM domains using the same peptides and the same solvent as for CD spectroscopy and DHX. Secondary chemical shifts are calculated as deviations from random coil chemical shifts of the respective amino acids. Negative Hα and positive Cα secondary chemical shifts that characterize α-helices reveal that the GnTV-TM domain is α-helical over the entire sequence, but that the helical conformation in the central part between G22 and G26 is slightly less pronounced compared to the neighbouring regions (Fig. 5; Supplementary Fig. 8). This finding is corroborated by the pattern of nuclear Overhauser effects (NOE), that reflect a typical α-helix, particularly because of the presence of a continuous stretch of Hαi-HN_i+3_ and Hα_i_-Hβ_i+3_ NOEs for most of the TM domain (Supplementary Fig. 9). The central α-helical part appears less structured as for example an NOE between G22 Hα and W25 Hβ is missing (Supplementary Fig. 9). ϕ/ψ Backbone angles and S^2^ order parameters (Supplementary Fig. 10) that report on helix stability were derived from chemical shifts with TALOS +^53^ and underscore this finding as well as NOE patterns and S^2^ analysis (Supplementary Figs. 9 and 10). The secondary chemical shifts and NOE data for the mutants indicate a stabilisation of the central part of the helix for the G22L and G22L/G26L mutant, and a loss of local helicity between T20 and P26 for G26P (Supplementary Figs. 8 and 9), especially as in the latter the characteristic Hα_i_Hβ_i+3_ NOEs between T20 and P26 are missing.

**Figure 6:**
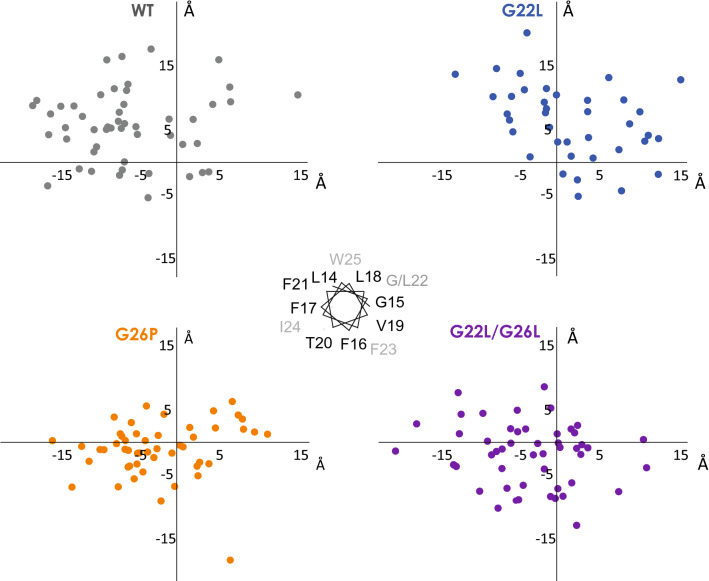
Spread of the structural bundles superimposed on L14-F21. Shown is the position of the C-terminal helix end (at residue 35) when the N-terminal is aligned along the z-axis thus pointing out of the plane of the figure. The position of residues L14–W25 are depicted by the central helical wheel.

Structure calculations confirmed that the GnTV-TM domain is helical over the entire TM domain region. The bundle of possible conformations is displayed superimposed on the N-terminal part covering residues 14–22 (Fig. 5). It fans out comparable to what we observed for the APP-TM domain^52^ and TNFα-TM domain^22^ so that the C-terminal ends lie within a cone. The direction of the resulting cone is not arbitrary in the GnTV-TM domain, as visualized by the positions of the C-terminal helix ends (at residue 35) and thus the spread of the structural bundles (Fig. 6). In this representation, the N-terminal helix (residues L14 to F21, depicted by the central helical wheel in Fig. 6) is aligned along the z-axis thus pointing out of the plane of the figure (Fig. 6). The ends of most of the 50 aligned GnTV WT structures can be found in the upper left quadrant. Bending into the direction of the upper quadrant was almost abolished in the mutants (Fig. 6). Proline is not able to form a hydrogen bond to G22 due to its missing amide hydrogen, and has a narrower range of accessible backbone angles. Apparently, this reduced the number of possible conformations exhibited by GnTV G26P. Interestingly, the double mutation G22L/G26L had a similar, albeit less pronounced effect (Figs. 5 and 6). In contrast, the G22L mutation changed mainly the direction of the bending and thus the orientation of the cone (Figs. 5 and 6).

Thus, the structural characterization shows, in accordance with the DHX data, that the TM helix of GnTV is destabilized in its central part and that—as predicted—this part becomes more helical by mutating both glycines to leucines, whereas the G26P mutation reduces helicity. Although the GnTV TM sequence does not contain an obvious hinge motif, like a double glycine motif in its centre, naively, one could have assumed that glycines G22 and G26 tolerate higher curvature along that side of the helix in analogy to the APP-TM domain^52^. However, the helical wheel (Fig. 6) shows that the concave side of the helix is rather found 100° counterclockwise where several aromatic residues (F17, F21, W25) are located (Fig. 6). The sidechains of F21 and W25 arrange in an offset stacked favourable interaction (Fig. 6^54^). Mutation of one or both of the adjacent glycines led to marked changes in the curvature. Incorporation of a leucine side chain on position 22 led to an altered curvature. The concave side of the helix is now found turned by ~ 100° around the helix axis, leucines on both positions, 22 and 26, turned the concave side by twice this amount, whereas proline on the second position furthermore restricted the possible space.

## Discussion

Although intramembrane proteases and their substrates have been subject to intensive research in the past decades, the general properties that define a *bona fide* substrate for intramembrane proteolysis remain enigmatic. While proteolytic cleavage requires the hydrolysis of a peptide bond and the scissile site to be part of an extended conformation^55^, TM domains are usually α-helical^56^ and the conformational flexibility of intramembrane protease substrates has been topic of various studies^22,25–27,30,47,52,57–62^.

This study explores the substrate requirements of the intramembrane aspartyl protease SPPL3 and demonstrates that introducing single amino acid mutations in the GxxxG motif of the GnTV TM domain affect its conformational flexibility, which results in altered SPPL3-dependent processing of this substrate. These alterations in substrate processing may be induced by changes in substrate affinity and/or changes in turnover rate. Bri2, a substrate of SPPL2b, also comprises a GxxxG sequence in the centre of its TM domain. In contrast to GnTV, neither of the two glycines was found to be of critical importance for the helical stability of the Bri2 TM domain, as suggested utilizing CD spectroscopy, and its cleavage by SPPL2b^27^. However, a thorough analysis of the conformational flexibility had not been implemented and only glycine-to-alanine substitutions had been investigated^27^. Since glycine-to-alanine substitutions also had only mild effects on intramembrane cleavage of GnTV (Fig. 1), the role of the GxxxG motif in context of Bri2 proteolysis cannot be fully evaluated so far. Interestingly, a glycine at the N-terminus of the Bri2 TM domain was mainly responsible for both a reduced helicity of the TM domain and efficient SPPL2b dependent cleavage^27^. GnTV also comprises an additional glycine N-terminally of the GxxxG motif (Fig. 1A). However, this glycine is positioned close to the annotated border of the TM domain and the intracellular juxtamembrane domain and, therefore, may not unequivocally be part of the actual TM domain. Instead, the first glycine of the GxxxG motif in GnTV maps to the region of the critical glycine within the Bri2 TM domain, this may suggest that helix-destabilizing amino acids at certain positions in the N-terminal half of the substrate’s TM domain facilitate cleavage by SPP/SPPL proteases.

Unfortunately, the ability to fully evaluate the effect of these GnTV TM domain mutations on SPPL3-dependent cleavage is limited in the current study due to the presence of unknown protease(s) involved in secreting sGnTV (Fig. 1C,D). The finding of other protease(s) being involved in the secretion of GnTV is not surprising. Since the substrate spectra of SPPL3 includes a wide variety of glycosidases and glycosyltransferases^23^, complete lack of the protein leading to total inhibition of their processing would have a detrimental effect on the organism. Thus, it can be expected that other proteases have evolved to support shedding and intracellular inactivation of these substrates, though possibly to a lesser extent. For some sialyltransferases, for instance, it has already been shown that BACE1 is capable of cleaving these enzymes^43–45^. To assess the contribution of SPPL3 to GnTV shedding, an indirect approach using SPPL3 KO cells was chosen (Fig. 1 and Supplementary Fig. 2). Analysis of SPPL3 KO cells revealed the relative share of the unknown protease(s) on GnTV processing and allowed calculation of the relative SPPL3 contribution (Fig. 1C,D). This revealed that GnTV G26P and G22L are cleaved by SPPL3 with similar efficiency as GnTV WT, while all other mutations showed reduced SPPL3-dependent cleavage, with GnTV G26L and G22/G26L having the strongest effect (Fig. 1D). However, this does not take into account that the total secretion of the mutants is also affected. For instance, the relative cleavage of GnTV G26P by SPPL3 and the unknown protease(s) is similar to that of GnTV WT (Fig. 1D), but its total secretion is significantly increased (Fig. 1B). This indicates that all proteases cleave this mutant with higher efficiency. However, it cannot be completely excluded that cleavage by either of the proteases is more efficient and decreases the pool of GnTV G26P available for the other protease. In case the unknown protease(s) would cleave GnTV G26P more efficiently, SPPL3 would have less substrate available and given the strong increase in total secretion and the overall high contribution of SPPL3 to cleavage, this would then still suggest that SPPL3 processes the mutant protein more efficiently than GnTV WT. This was also supported by overexpression of SPPL3 WT that resulted in a stronger increase in secretion of GnTV G26P compared to secretion of GnTV WT, while secretion of GnTV G26L and G22/G26L was rescued by overexpression of SPPL3 WT but did not further increase secretion (Fig. 2B). In contrast, rescue of the SPPL3 KO with a catalytically inactive mutant did not change sGnTV secretion compared to the knockout condition (Fig. 2B). Demonstrating that changes in GnTV secretion are due to the catalytic activity of SPPL3 and are not mediated by CRISPR/Cas9 off target effects or non-catalytic functions of SPPL3. Consequently, the methods applied show the correct tendency of SPPL3-dependent processing but may underestimate the quantitative contribution of SPPL3. To solve this shortcoming of the method an in vitro assay utilizing purified substrate and protease would be required. However, due to the complicated architecture and the hydrophobic nature of the proteases all efforts to establish such an assay have failed so far.

MALDI-TOF mass spectrometry indicates that cleavage sites of SPPL3 in GnTV are not affected by mutations of the glycine residues within the GxxxG motif (Fig. 3). However, the relative intensity of the peaks reflecting the cleavage of the unknown protease(s) outside the GnTV TM domain (Q36) and the major cleavage of SPPL3 within the TM domain (L29) only partially match the quantitative results obtained from Western Blot analysis. While concordantly the SPPL3 dependent cleavage of GnTV G22L and G22L/G26L is reduced and, thus, hardly detectable in mass spectrometry, the major SPPL3 dependent (L29) cleavage of GnTV G26P seems also decreased relative to GnTV WT (Fig. 3). This may be attributed to different detection sensitivity of the fragments in MALDI-TOF mass spectrometry since the fragment terminating at L29 is longer and more hydrophobic than that terminating at Q36. In addition, GnTV G26P is cleaved better by both, SPPL3 and the unknown protease(s) (Fig. 1). Based on that the amount of Q36 peptide is expected to be produced in higher amounts and given its increased hydrophilic character and decreased length compared to the L29 peptide it is expected to be detected more easily by MALDI-TOF mass spectrometry. This may explain the unexpected ratio between Q36 and L29 peptides in mass spectrometric analysis of the GnTV G26P mutant. Furthermore, a possible direct or indirect contribution of SPPL3 to the production of the Q36 peptide might be considered, which could be reflected in the reduced intensity of the Q36 peak in SPPL3 KO cells compared to control cells (Fig. 3) that is not detected for the mutants G22L and G22L/G26L, which are primarily cleaved by the unknown protease(s). Both GnTV WT and G26P present a decrease in the intensity of the Q36 peak in the SPPL3 KO cells, which is more pronounced in GnTV G26P. This may either suggest that SPPL3 produces a percentage of the fragment starting at position Q36, if the flexibility of the GnTV TM reaches a certain level or that SPPL3 directly or indirectly acts on the unknown protease(s).

Using DHX we were able to show that the glycine residues forming the GxxxG motif in GnTV do indeed cause considerable instability of the TM helix (Fig. 4). Interestingly, mutations of the glycine residues not only resulted in local stabilization or destabilization but also affected more distant C-terminal regions of the GnTV TM domain around the actual SPPL3 cleavage sites. This agrees with recent findings on the non-canonical shedding of TNFα by SPPL2a^22^. Insertion of proline or leucine residues in the N-terminal half of the TNFα TM domain resulted in destabilization or stabilization, respectively, of the C-terminal TM part, where the initial SPPL2a cleavage sites are localized^22^. Similar to the G26P mutation in GnTV (Fig. 1), S34P in TNFα resulted in increased H-bond flexibility and SPPL2a mediated cleavage, while mutations to leucine in the TM domain of TNFα stabilized the helix of the TM domain and reduced SPPL2a mediated non-canonical shedding^22^. In agreement with these earlier findings, the stabilization of the GnTV TM domain by glycine to leucine mutations, as reported here (Fig. 4), resulted in reduction of SPPL3 dependent cleavage (Figs. 1 and 3).

Moreover, DHX measurements (Fig. 4) indicate pronounced conformational flexibility of the GnTV WT TM helix, where amides close to the centre of the helix exchange as rapidly as the amides of the frayed helix termini, despite being part of helical secondary structure, as documented by CD and NMR spectroscopy. This attests to exceptionally low-H-bond stability at G26 and about two helical turns downstream. Amides at many rapidly exchanging positions exhibit biphasic DHX suggesting that exchange after transient single and double H-bond openings can occur in parallel, thus again underscoring the pronounced flexibility of GnTV TM domain. It is likely, that the low stability of the WT GnTV TM helix originates from the presence of two glycines within the GxxxG motif. Mutating these glycine residues alters exchange rates at and around the mutated positions. G26P enhances DHX, in contrast, introducing Leu at positions 22 (GnTV G22L) and/or 26 (GnTV G22L/G26L) stabilizes the helix to various extents, as expected from the known impacts of the respective amino acids on overall helix stability^39^ and local flexibility^40,42^. Biphasic DHX kinetics was also detected at several sites within the Xbp1u TM helix and at a number of residues of the slightly bent TNFα TM helix, including its central A_42_G_43_A_44_ motif (our unpublished observations). Our NMR data reveal that the flexible area in the centre of the GnTV TM domain is surrounded by two more stable α-helical parts and suggested that mutations in the GxxxG motif may not only affect stability but also the possible mutual orientations of the N- and C-terminal α-helical parts. The correlations of the effect of the mutations on cleavability and structure suggest that the movement of those two α-helical parts relative to each other impacts on cleavage of the substrate. This effect of conformational dynamics has also been seen in other substrates of intramembrane proteolysis^22,47,62^. However, the different structures presented in the bundles are not a measure of true dynamics. Rather we show the plethora of possible orientations that all comply with the experimental data. It is feasible and highly likely that lateral pressure exerted by lipids or the interaction with the enzyme influence and maybe change this mutual orientation. However, it is unclear to which extent the intrinsic flexibility of the helices reflected by the geometries of the different structural ensembles may exert resists to those external constraints. The GnTV G22L mutation, which results in strongly reduced SPPL3 cleavage, changed the curvature orientation by about 30° (Figs. 5 and 6). The double mutation G22L/G26L led to a marked reduction in possible conformational space. Bending along the helix face where F21 and W25 side chains are found, was almost abolished in this mutant. Instead, the ends of the structural bundles lie within an elongated ellipsoid. The conformational space was even more restricted in G26P than in G22L/G26L (Figs. 5 and 6). A similar impact was also observed for mutations of a di-glycine hinge in the TM domain of APP^52^. A single glycine mutation (G38L) resulted in different mutual orientations of the two α-helical parts and reduced bending of the entire TM domain. In contrast, exchange to a proline led mainly to altered cone opening. APP-TMD mutants with altered bending were cleaved less efficiently by secretase^62^, which led to the speculation that a movement at a central hinge position may be required for substrate engulfing by the protease^52,62^. Notch1-TMD does not have a glycine hinge in its sequence but a stretch of four alanines. It adopted a straight and regular α-helix in DMPC/DHPC bicelles^63^, but the same region was somewhat distorted in the cryo-EM structure of the γ-secretase complex albeit without a bend in the flexible central α-helical parts^17^. DHX data suggested a hinge function of the tetra-alanine motif^48^. Exchange of the central AGA motif in TNFα to three leucines also reduced conformational space in the superimposed bundle and stabilized the central region^22^. In other words, a flexible substrate TM helix may be required for its translocation from a site of initial contact at the membrane-enzyme boundary towards internally located catalytic residues. Alternatively, a certain helical flexibility may also promote unfolding of the substrate at the cleavage site. A requirement for unfolding at the cleavage site is well established in case of soluble protease substrates where the region around a scissile bond is docked onto the protease’s active site in an extended conformation^64^. Accordingly, the scissile bonds of soluble substrates tend to be abundant in loop regions and in unstable helices^65^. Partial unfolding of the helix around the initial cleavage sites was also shown for the TM domains of the γ-secretase substrates Notch1^17^ and APP^17^. In the case of GnTV, the most flexible region downstream of G26 overlaps with the major SPPL3 cleavage site between M28 and L29. Therefore, our data are compatible with both scenarios in GnTV cleavage by SPPL3. Nonetheless, the structural relationship between SPP/SPPL enzymes and presenilin, the enzymatic subunit of γ-secretase, as well as mechanistic considerations suggest that it is the translocation of the substrate TM domain to the catalytic residues of an intramembrane protease that is supported by bending motions of the TM-helix. Notably, substrate processing by γ-secretase occurs within minutes to hours and is characterized by extremely low catalytic constants^66,67^. Accordingly, the combined process of substrate translocation, cleavage, and product release is slow. One might expect that SPP/SPPL-type proteases are slow enzymes, too, and it might be assumed that the rate-limiting step in GnTV processing corresponds to slow translocation of a flexible substrate TM helix to the catalytic cleft. By contrast, local helix unfolding at the cleft is likely to occur within milliseconds upon arrival of the TMD within the aqueous environment at the catalytic cleft, as suggested by studies on soluble helices^68^. That a flexible region in a TM helix can influence cleavage of distant bonds by an SPPL protease is demonstrated by TNFα TMD cleavage by SPPL2a. There, stabilizing a central helix bend mediated by an AGA motif by mutation impaired cleavage at a downstream site. By contrast, helix-destabilizing mutations directly at the cleavage site did not enhance cleavage^22^. Thus, the pronounced flexibility of the GnTV TM helix might primarily promote the translocation of a substrate towards the catalytic cleft of SPPL3.

Overall, the GnTV WT TM domain depicts an extraordinary flexibility compared to other intramembrane substrates^22,30,47,52,62,63^, which is mainly determined by a GxxxG motif. Considering many data collected on the substrate requirements of intramembrane aspartyl protease substrates to date, it turns out that the requirement for a certain flexibility of the helix seems to be common for many if not all substrates. However, the degree of flexibility that is required for efficient cleavage seems to vary between different substrate enzyme combinations. Although other domains, like juxtamembrane domain, may also contribute substrate processing by intramembrane proteases, the helix flexibility of substrate’s TM domain seems to play a major role. Comparative future studies on different substrate-enzyme combinations will be helpful to establish a more complete understanding of substrate requirements in recognition and cleavage by intramembrane proteases.

In summary, our data provide evidence that increased helix flexibility of the TM domain promotes SPPL3 mediated cleavage of GnTV, while decreased helix flexibility has the opposite effect. This suggests that *bona fide* SPPL3 substrates require to have certain helix flexibility in their TM domains that allows entry of the substrate to the catalytic cleft. This understanding will help to more easily predict substrates and non-substrates of SPPL3 using bioinformatics and, thus, will allow to broaden our understanding of SPPL3 in regulating cellular glycosylation and its potential impact on disease development.

## Materials and methods

### Antibodies

Monoclonal antibodies against SPPL3 (clone 7F9), were characterized previously^20^. Anti-V5 (mIgG2A, clone R960-25, Life Technologies, Carlsbad, USA), anti-Calnexin (rabbit pAb, Enzo Life Sciences, Farmingdale, USA), anti-TGN46 (sheep, pAb, AP32693PU, Acris, Maryland, USA). Horseradish peroxidase (HRP)-conjugated secondary antibodies were purchased from Promega (Madison, USA), and the fluorescent secondary antibodies donkey anti-mouse 488 and donkey anti-sheep 568 were obtained from Thermo Fisher, Waltham, USA.

### Generation of SPPL3 knock out cells

Two guide RNAs targeting the *SPPL3* gene were purchased from Sigma-Aldrich (Darmstadt, Germany; HS0000515665, HS0000515666). T-Rex™-293 (Thermo Fisher, Waltham, USA) (HEK293) cells were transiently transfected with either one of the guide-RNAs with a pCMV-Cas9-GFP gRNA plasmid and one day after transfection, the GFP expression was checked using an LSM 710 Zeiss Observer Z.1 confocal microscope to confirm the transfection’s efficiency. Two days post transfection the cells were subjected to single-cell FACS-sorting selecting for GFP positive signal. The single cell clones were propagated and the expression levels of SPPL3 were analyzed for each clone by Western Blotting. Clones with no SPPL3 levels detectable underwent genomic DNA sequencing to confirm the presence of a homozygous mutation leading to an early stop codon.

### cDNA constructs

The cDNA that encodes human *MGAT5* (protein name: GnTV) with a N-terminal Flag (DYKDDDDK) epitope tag after the initiating methionine residue and a C-terminal in-frame V5 (GKPIPNPLLGLDST) epitope tag (Flag- GnTV-V5) was described before^21^. The mutated GnTV constructs were cloned via Quick Change and the primers were designed using the (http://www.genomics.agilent.com/primerDesignProgram.jsp) online software by Agilent Technologies. The cDNA constructs used for the Mass Spectrometry analysis, were designed using the Flag-GnTV-V5 as template. A second Flag tag (DYKDDDDK) was inserted after the TM domain at position 53 of the GnTV protein, followed by a TEV cleavage sequence (ENLYFQG) and the rest of the GnTV sequence. All primer sequences are available upon request, and all expression constructs were sequence-verified prior to experimental use.

### Cell culture, transfection, protein extraction and immunoblotting

T-Rex™-293 (Thermo Fisher, Waltham, USA) were maintained in DMEM GlutaMAX™ media (Thermo Fisher, Waltham, USA) supplemented with L-glutamine (Life Technologies, Carlsbad, USA), 10% (v/v) fetal calf serum (Sigma-Aldrich, St. Louis, USA), 1% (v/v) penicillin/streptomycin (Life Technologies, Carlsbad, USA), and 5 μg/ml blasticidin (Life Technologies Carlsbad, USA). Transient cDNA transfections were achieved using Lipofectamine™ 2000 according to the manufacturer’s instructions. To establish stable transfection transient transfection was followed by 100 μg/ml hygromycin (Life Technologies, Carlsbad, USA) antibiotic selection. Afterwards cells were kept in presence of 100 mg/ml hygromycin. SPPL3 WT and SPPL3 D272A expressing cells were generated by stably transfecting the respective cDNAs into doxycycline-inducible TR SPPL3 KO cells and keeping the cells in the presence of zeocin. Expression of the SPPL-proteases was induced by 1 mg/ml of doxycycline in the culture medium for 48 h.

To detect secreted GnTV, cells were incubated in the same media for 24 to 48-h and conditioned media was collected. The media was then cleared at 17,000 g, 4 °C for 20 min. For detection of intracellular proteins, samples were kept on ice for the whole protein extraction. First the cells were washed with ice-cold PBS (140 mM NaCl, 10 mM Na_2_HPO4, 1.75 mM KH_2_PO4, pH 7.4), harvested and then lysed with ice-cold STE buffer (150 mM NaCl, 50 mM Tris, pH 7.6, 2 mM EDTA), freshly supplemented with 1:500 protease inhibitor mix (Sigma-Aldrich, St. Louis, USA). Samples were incubated on ice for 30 min, and cell debris was removed by centrifuging at 17,000 g, 4 °C for 30 min. To correct for inconsistencies between sample concentrations, values were normalized based on the total protein concentration in the lysates, which was determined using a BCA assay (Interchim, Montlucon, France).

For detection of secreted or intracellular proteins, conditioned media or cell lysates were subjected to sodium dodecyl sulfate polyacrylamide gel electrophoresis (SDS-PAGE) analysis. The proteins were separated on an acrylamide gel and transferred onto polyvinylidene fluoride (PVDF) membranes (PVDF; immobilon P transfer membrane, 0.45 mm pore width; Millipore). The proteins were then detected as described previously^69^.

For statistical purposes, three biological replicates were used for each experiment. Each biological replicate was seeded and analyzed on a different week, and each consisted of three technical replicates. For the statistical analysis, n = 3 refers to the three biological replicates.

### Immunostaining and confocal microscopy

Stably transfected cells were used for imaging and cells were seeded at a confluency of ~ 30% on 10-mm Poly-L-Lysine coated coverslips. Two days post-seeding the media was removed, cells were washed once with PBS and then fixated for 20 min at room temperature in 4% paraformaldehyde in PBS. Cells were permeabilized for 5 min using 5% (w/v) bovine serum albumin (BSA) supplemented with 0.1% (v/v) Triton X-100 and then blocked by one-hour treatment with 5% (w/v) BSA at room temperature. For detection of the proteins, cells were incubated with primary antibodies diluted in 5% (w/v) BSA for 2 h at room temperature, followed by secondary antibodies diluted 1:400 in 5% (w/v) BSA for one hour at room temperature. Finally, cells were incubated for 5 min with 1:10,000 40,6-Diamidino-2-phenylindol (DAPI, 5 μg/ml) solution at room temperature to visualize the cell nuclei. Coverslips were loaded onto glass plates using ProLong Glass Antifade Mountant (Thermo Fisher, Waltham, USA). Pictures were taken using a LSM 800 Zeiss Observer Z.1 confocal microscope with a 60 × oil lens and 2 × optical zoom. The intensity of the pictures was increased for visualization purposes with the Zen lite 2011 program.

### Immunoprecipitation and Mass Spectrometry

For detection of cleavage sites in sGnTV, cells were transfected with GnTV mutants comprising the TEV cleavage site. Three days post transfection the conditioned media was collected and cleared at 17,000 g, 4 °C for 20 min. Anti-Flag® M2 affinity agarose beads (Sigma-Aldrich, Darmstadt, Germany) were used to precipitate secreted GnTV, 2 h at room temperature, and the isolated peptides were eluted from the beads with 100 mM glycine pH 2.5. The eluted peptides were then cleaved with AcTEV Protease overnight according to the manufacturer’s instructions (Thermo Fisher, Waltham, USA). The TEV cleaved peptides were then precipitated with anti-Flag® M2 affinity agarose beads for 1 h followed by 3 washes with MS washing buffer (0,14 M NaCl, 0,1% N-octyleglycopyranoside, 10 mM Tris-HCl pH 7,6, 5 mM EDTA) and 2 washes with dH2O. Samples were analyzed by mass spectrometry using α-cyano-4-hydroxycinnamic acid matrix (Sigma Aldrich, Darmstadt, Germany) mixed with acetonitrile and 0,6% TFA (1:1 ratio). 1.2 μl of sample were spotted on a 384 spot plate and dried at room temperature. Mass spectra were recorded on a 4800 MALDI TOF/TOF analyzer (Applied Biosystems, Hennigsdorf, Germany) in the linear mode with external calibration.

### Peptide synthesis for DHX

Peptides for DHX comprising amino acid 13–37 of GnTV were synthesized by Fmoc chemistry (PSL, Heidelberg, Germany). Two lysines were added onto each terminus and the termini were blocked by acetylation and amidation of termini, resulting in the wild-type TMD sequence Ac-KK KLGFFLVTFGFIWGMMLLHFTIQQRKK-amide. The peptides were purified by HPLC. Purity was > 90% as judged by mass spectrometry. Concentrations were determined via UV spectroscopy using an extinction coefficient at 280 nm of 5500 M^-1^·cm^-1^. All other chemicals were obtained from Sigma-Aldrich Co and Honeywell Fluka™.

### Circular dichroism spectroscopy

For CD spectroscopy, peptides were dissolved at 50 µM in 80% (v/v) TFE in 5 mM PBS, pH 7.4. CD spectra were obtained using a Jasco J-710 CD spectrometer from 190 to 260 nm in a 1.0 mm quartz cuvette at 20 °C with a response of 1 s, a scan speed of 100 nm/min and a sensitivity of 100 mdeg/cm. Spectra reflect signal-averaged accumulations of 10 scans with the baselines (corresponding to solvent) subtracted. Mean molar ellipticities were calculated and secondary structure contents estimated by deconvoluting the spectra using CDNN software^70^ with CDNN complex reference spectra.

### Deuterium/Hydrogen exchange (DHX) experiments and Electron Transfer Dissociation (ETD) D/H exchange

Prior to DHX and Electron Transfer Dissociation D/H-exchange (ETD-DHX) peptides were deuterated in 80% (v/v) d_1_-HFIP in D_2_O at a concentration of 300 μM and incubated at 37 °C for 7 days. To avoid contamination with dissolved polypropylene from reaction tubes, glass inserts were used in 1.5 ml safe-lock tubes. After 7 days, the solvent was removed by SpeedVac centrifugation. The remaining peptide pellet was dissolved in 80% (v/v) d_1_-TFE in 2 mM ND_4_-acetate in D_2_O. Deuteration level was verified by mass spectrometry to be > 95%.

For global DHX measurements, the deuterated peptides were diluted 1:20 from a 100 μM deuterated peptide stock solution in 80% (v/v) TFE in 2 mM NH_4_-acetate, pH 4.0 (peptide final concentration: 5 μM) and incubated for different time periods (t = 1 min, 2 min, 5 min, 10 min, 30 min, 1 h, 2 h, 4 h, 8 h, 16 h, 24 h, 48 h, 72 h, 7 d) in 0.5 ml Eppendorf safe-lock tubes (Eppendorf, Germany) at 20.0 °C in a thermal cycler (MasterCycler from Eppendorf, Germany). To slow down the exchange reaction before measurement, samples were quenched by putting them on ice and adding formic acid (0.5% (v/v) final concentration) which lowered the pH to ≈2.5. Mass/charge (m/z) ratios were recorded after the indicated time periods using a Synapt G2 HDMS mass spectrometer (Waters Co., Milford (MA), USA) with one scan/second.

For ETD-DHX measurement, a solution of deuterated peptide (100 μM) was diluted 1:20 with protonated solvent (80% (v/v) TFE in 2 mM NH4-acetate, pH 4.0 (standard pH) to a final peptide concentration of 5 μM and incubated at 20 °C in a thermal cycler. Different incubation times (t = 0.1 min, 1 min, 2 min, 5 min, 10 min, 30 min, 1 h, 2 h, 4 h, 8 h, 16 h, 24 h, 48 h, 72 h, 7 d, 10 d, 20 d) were applied. ETD was conducted by injecting the cooled and quenched peptide samples with a 100 μl gas-tight Hamilton syringe (5 μl/min) and a short PEEK-tubing with a needle port. During the measurement, the syringe was cooled down by a − 20 °C cool pack. The ETD MS /MS measurements were carried out in sensitivity mode. As an ETD reagent, 1,4- dicyanobenzene was used, which was delivered by a flow of nitrogen gas (makeup flow: 30 ml/min). The glow discharge ion source was operated at 60 μA, with refill times of 100 ms between 1 s MS/MS scans. The selection window for MS/MS mode was ± 2.0 m/z units. The trap T-Wave ion guide was operated with a wave height of 0.2 V and a wave velocity of 300 m/s. Spectra were measured over 10 min in the range of m/z 50–3500 by selecting the 5 + charged peptides as MS/MS precursor for ETD. Further parameters were: capillary voltage (2.8 kV), sampling cone (20–22 V), extraction cone (2.0 V), source temperature (90 °C), desolvation gas temperature (300 °C), cone gas (0 L/h), desolvation gas (800 L/h), transfer collision voltage (5.0) and gas control (trap 14 ml/min and transfer 0.3 ml/min). ETD was started by decreasing the wave height voltage from 1.5 to 0.2 V. All experiments were done at least in triplicate. For ETD spectra data evaluation, the ETD Fragment Analyzer module of the software suite MassMap® (MassMap GmbH & Co. KG, Wolfratshausen, Germany) was used^47^. It is based on GRAMS/AI (Thermo Fisher Scientific, Waltham (MA), USA). ETD spectra over 10 min were combined and evaluated.Monoexponential fitting of the data was done with Eq. (1) to calculate k_exp,DHX_, which accounts for the concentration of deuterated solution in the DHX-ETD assay of 5% (v/v).

1 $$\mathrm{D}(\mathrm{t})=0.95^*{\mathrm{e}}^{\wedge}{({(-{\mathrm{k}}\_{({\mathrm{exp},\mathrm{DHX}})}. \mathrm{ t})})}+0.05$$

while biexponential fitting was done with Eq. (2):

2 $$\mathrm{D}\!\left(\mathrm{t}\right)=\mathrm{A}. {\mathrm{e}}^{\wedge}{({(-{\mathrm{k}}\_{({\mathrm{exp},\mathrm{DHX},\mathrm{A}})}\mathrm{t})})}+\mathrm{B}. {\mathrm{e}}^{\wedge}{({(-{\mathrm{k}}\_{({\mathrm{exp},\mathrm{DHX},\mathrm{B}})}\mathrm{t})})}+0.05$$

where A and B are the population sizes of the deuterons with slower and faster exchange rates, k_exp, DHX, A_ and k_exp, DHX, B,_ respectively and A + B = 0.95.

Residue-specific DHX kinetics (Eqs. (1) and (2)) originate from time-dependent deuteron contents D_mean_ averaged from the corrected masses of different fragment ions. In brief, the rate constants k_exp, DHX_ were determined by a non-linear least square fitting routine. Standard errors of logk_exp,DHX_ result from the errors of the fits. The limits of the standard confidence interval of ΔG are calculated by means of the standard error of logk_exp,DHX_.

### NMR spectroscopy

Peptides used were identical to those used for DHX. Dry peptides were dissolved in 500 µL TFE-d2/H_2_O (80/20) to a final concentration of 500 µM. pH values were adjusted to 7.0. A set of homo- and heteronuclear NMR spectra was acquired on a 600 MHz Bruker Avance III spectrometer equipped with a TCI cryo-probe (Bruker BioSpin, Rheinstetten, Germany). For acquisition and spectral processing TopSpin (Bruker BioSpin, Rheinstetten, Germany) was used.

For assignment structure calculation ^1^H^1^H-TOCSY, ^1^H^1^H-NOESY, ^1^H^13^C- and ^1^H^15^N-HSQC spectra were measured. Resonances were assigned and peaks were integrated with CCPNMR^71^.

Secondary chemical shifts were determined as difference between measured and random coil values^72^. Backbone dihedral angles and S^2^ order parameters were predicted based on chemical shift values with TALOS +^53^. Structures were calculated on these dihedral angles and distances restraints derived from NOESY peak intensities with Aria2^73^. The atomic coordinates and experimental data have been deposited in the Protein Data Bank (www.wwpdb.org) and the Biological Magnetic Resonance Data Bank (BMRB, https://bmrb.io/): 7YYI / 34710 (WT), 7Z08 / 34712 (G22L), 7Z0B / 34713 (G22L/G26L), 7Z07 / 34711 (G26P).

All structures were first superimposed on L14–F21 in pymol. For turning the structures along the z-axis, the helix axes were determined from the local centroids of Cα tetrapeptide units of the helix using a python script.

## Supplementary Information

Supplementary Information.

## Data Availability

The NMR data sets (atomic coordinates and experimental data) generated during this study experimental data have been deposited in the Protein Data Bank (www.wwpdb.org) and the Biological Magnetic Resonance Data Bank (BMRB, https://bmrb.io/): 7YYI / 34710 (WT), 7Z08 / 34712 (G22L), 7Z0B / 34713 (G22L/G26L), 7Z07 / 34711 (G26P). The mass spectrometry data related to the DHX kinetics experiments generated during this study, are available at Mendeley Data Repository, https://doi.org/10.17632/9pfkykkwv7.1. Cell lines and cDNA constructs generated in this study will be made available on request, but we may require a payment and/or a completed Materials Transfer Agreement if there is potential for commercial application. Further information and requests for resources and reagents should be directed to and will be fulfilled by Regina Fluhrer (regina.fluhrer@med.uni-augsburg.de).
