# Supplementary material for: Helical stability of the GnTV transmembrane domain impacts on SPPL3 dependent cleavage
Source: Sci Rep. 2022 Dec 5;12:20987. doi: 10.1038/s41598-022-24772-8 (PMC9722940; doi:10.1038/s41598-022-24772-8)
Supplement: Supplementary file 1 — Supplementary Information. [file 41598_2022_24772_MOESM1_ESM.pdf]

Helical stability of the GnTV transmembrane domain impacts on SPPL3 dependent cleavage

Alkmini A Papadopoulou[1], Walter Stelzer[2], Mara Silber[3], Christine Schlosser[1], Charlotte Spitz[1], Martina Haug-Kröper[1], Tobias Straub[4], Stephan A Müller[5], Stefan F Lichtenthaler[5,6,7], Claudia Muhle-Goll[3], Dieter Langosch[2] and Regina Fluhrer[1*]

[1]Biochemistry and Molecular Biology, Institute of Theoretical Medicine, Faculty of Medicine, University of Augsburg, Universitätstrasse 2, 86159 Augsburg, Germany
[2]Lehrstuhl für Chemie der Biopolymere, Technische Universität München, Weihenstephaner Berg 3, 85354 Freising, Germany
[3]Karlsruhe Institute of Technology, Institute for Biological Interfaces 4, 76344 Eggenstein-Leopoldshafen, Germany and Karlsruhe Institute of Technology, Institute of Organic Chemistry, 76131 Karlsruhe, Germany
[4]Core Facility Bioinformatics, Biomedical Center, Ludwig Maximilians University Munich, 82152 Planegg-Martinsried, Germany
[5]DZNE – German Center for Neurodegenerative Diseases, Munich, Germany
[6]Neuroproteomics, School of Medicine, Klinikum rechts der Isar, Technical University of Munich, 81675 Munich, Germany
[7]Munich Cluster for Systems Neurology (SyNergy), Munich, Germany

*Address correspondence to
Regina Fluhrer, Biochemistry and Molecular Biology, Institute of Theoretical Medicine, Medical Faculty, University of Augsburg, Universitätsstrasse 2, 86159 Augsburg, Germany, Tel.: +49 (0) 821 598 – 2069, regina.fluhrer@med.uni-augsburg.de

Supplementary Material

Supplementary Figure 1

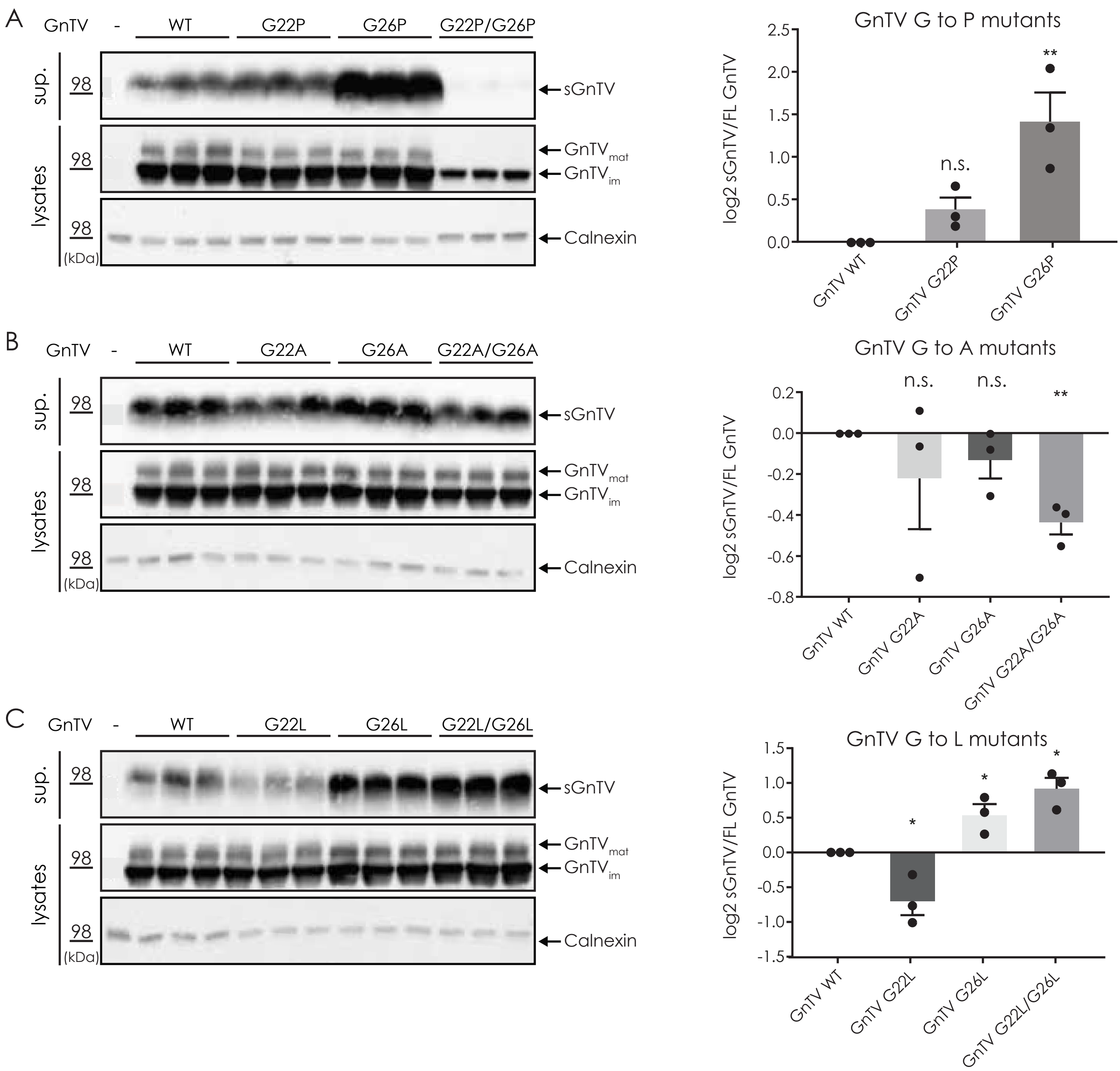

Supplementary Figure 1 Cleavage of GnTV GxxxG mutants. HEK293 cells ectopically expressing GnTV WT and the indicated GxxxG mutants were analyzed for secretion of soluble GnTV (sGnTV) detected in the conditioned media (sup.). **A.** Glycine to Proline mutants **B.** Glycine to Alanine mutants **C.** Glycine to Leucine mutants. Full length GnTV is detected in the lysates using the V5 monoclonal antibody in a mature ($GnTV_{mat}$) and an immature form ($GnTV_{im}$). For quantifications, the amount of sGnTV was divided by the amount of full-length GnTV (FL GnTV), comprising mature ($GnTV_{mat}$) and immature ($GnTV_{im}$) GnTV. The secretion was normalised to that of GnTV WT, which was set to 1 and the log2 for each mutant was calculated. Western Blots represent one biological replicate, each mutant comprising three technical replicates. Three biological replicates were included in the calcualtions refleceted in the bar graphs on the right side (mean + SEM). Multiple unpaired, two-tailed t tests with Holm-Sidak multiple comparisons correction n.s.= non significant, *p<0.05, **p<0.01.

Supplementary Figure 2

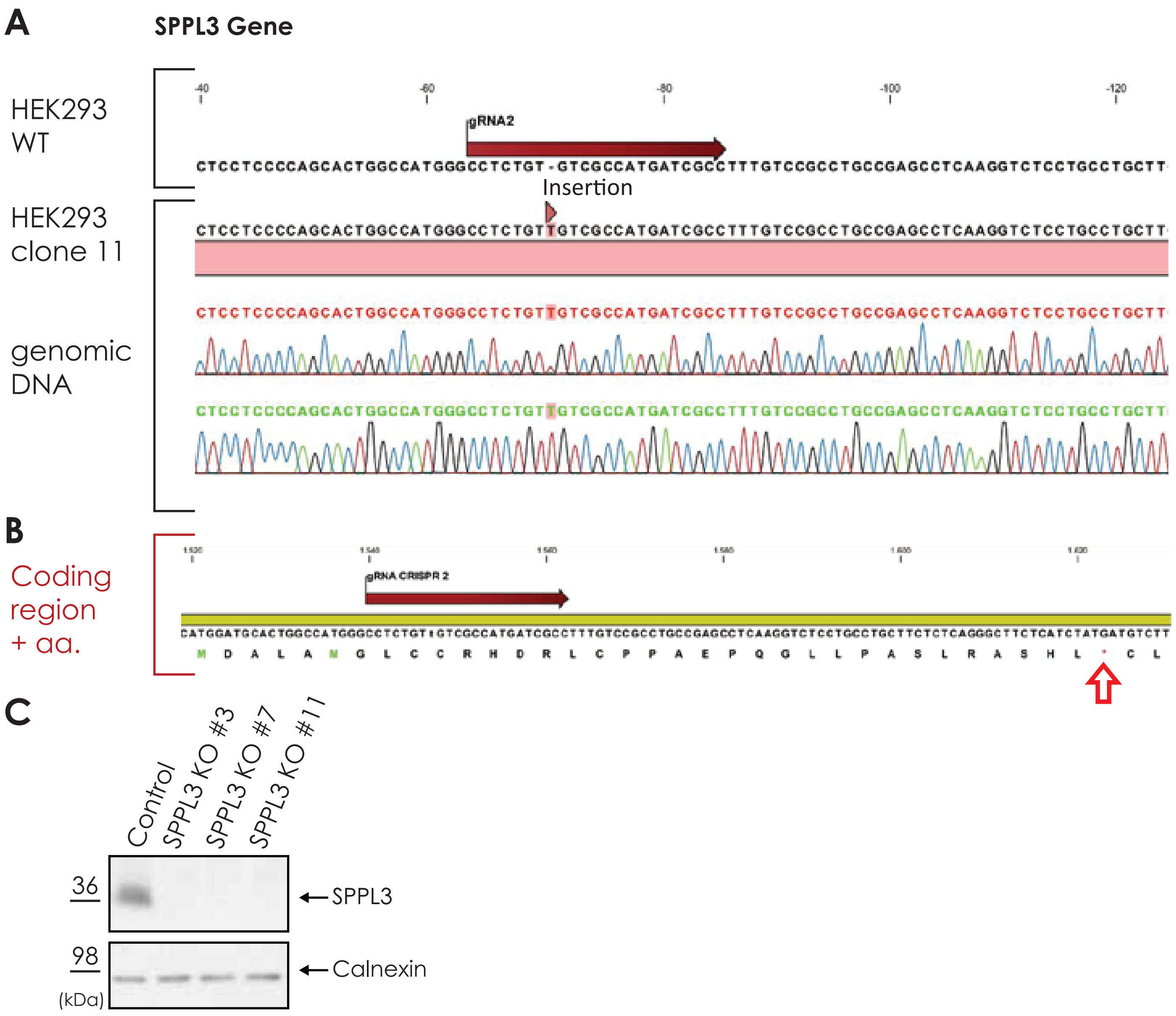

Supplementary Figure 2: HEK293 SPPL3 knockout (KO) cell line. **A.** Comparison of the wildtype human SPPL3 genomic DNA with KO clone 11 is depicted confirming the presence of homozygous insertion of a thymidine, highlighted in red. **B.** Protein coding sequence of clone 11 with the thymidine insertion and the amino acid sequence resulting from this nucleotide sequence. An early stop-codon is generated as indicated by the red arrow. **C.** Western blot analysis of control HEK293 cells and the three SPPL3 KO clones deriving from the CRISPR/Cas9 treatment, demonstrating the absence of SPPL3 protein expression. Calnexin was used as a loading control.

Supplementary Figure 3

A

Flag GnTV Flag TEV

DYKDDDDK ALFTPWKLSSQKLGFFLVTF**G**FIW**G**MMLLHFTIQQRTQPESSSMLREQILDLS DYKDDDDK ENLYFQ

B

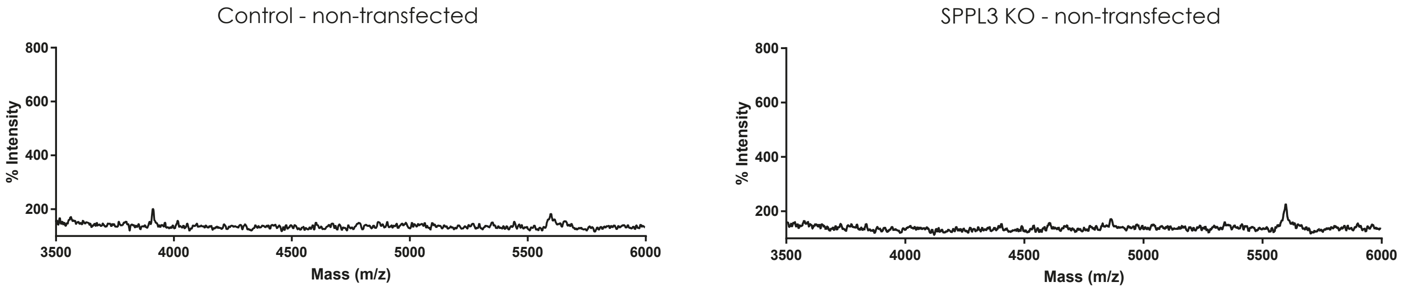

C

C-peptide (cleavage position)	Predicted (in Da)	Measured (in Da)
29	4774	4778
30	4661	4666
31	4547	4552
33	4263	4266
36	3903	3907

Supplementary Figure 3

A. Amino acid sequence and color coded representation of Flag-GnTV-FlagTEV peptide used in Figure 3. Flag tags in green, TEV cleavage site in purple and the major SPPL3 cleavage site indicated with red arrow **B.** Non-transfected controls of Mass Spectrometry experiment from Figure 3 **C.** Table of predicted and measured GnTV peaks. Numbers are the same for WT and mutants as the mutations are not in the peptides measured.

Supplementary Figure 4

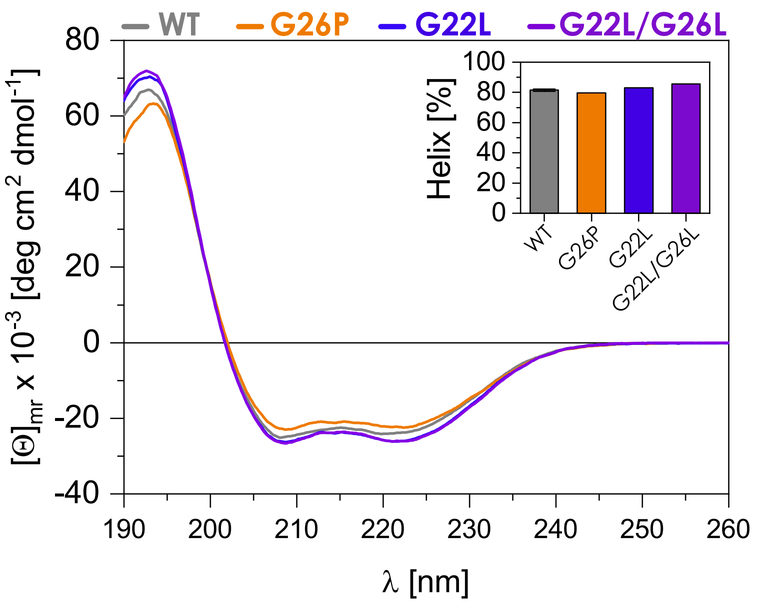

Supplementary Figure 4. Secondary structure of GnTV WT and mutant TM domain peptides determined by circular dichroism spectroscopy. Spectra were recorded in 80% TFE, 5 mM phosphate buffer pH 7,4 (averaged spectra). Inset: Calculated helix contents with CDNN (complex reference spectra), n= 2, means (± SEM)

Supplementary Figure 5
part 1

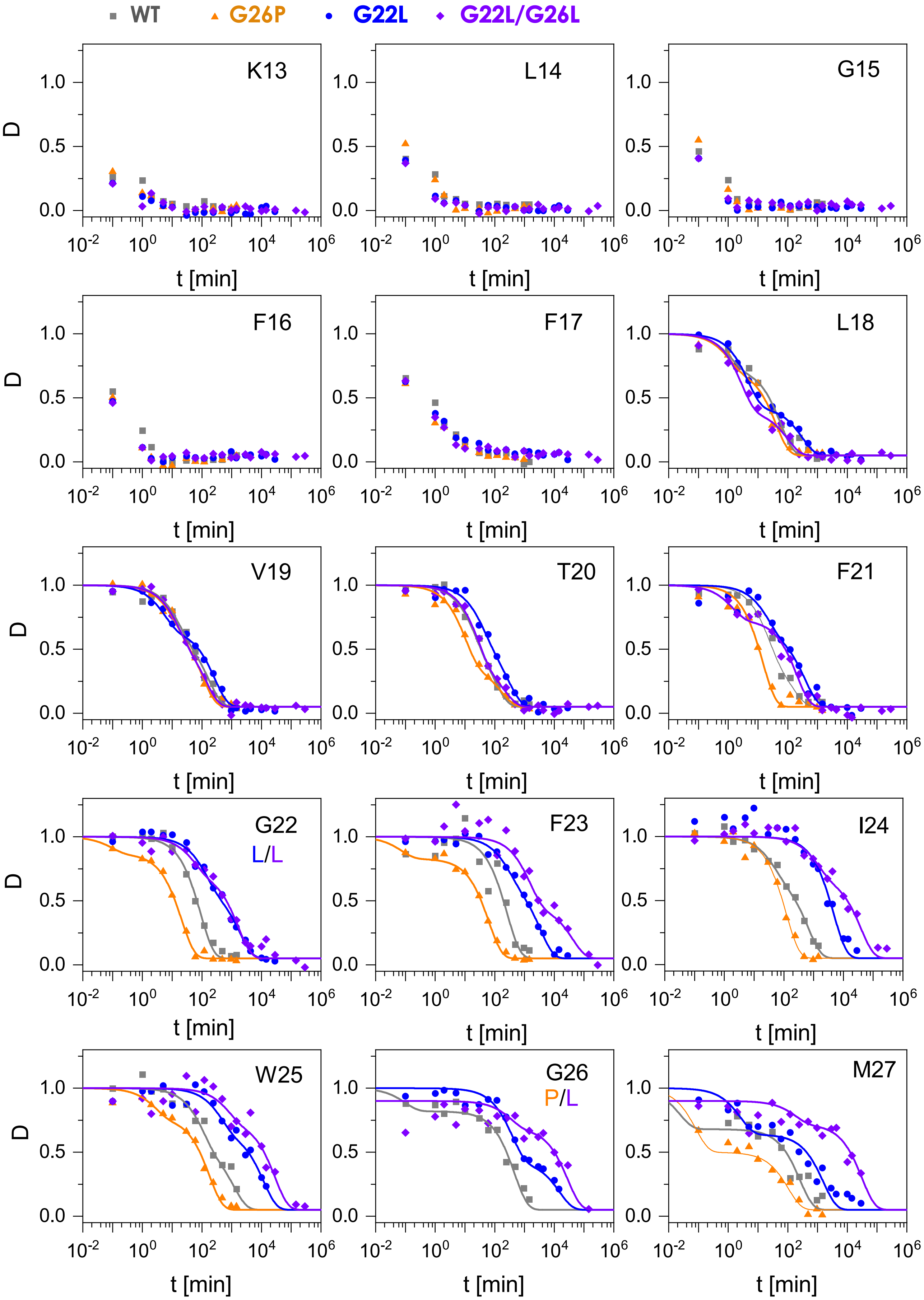

Supplementary Figure 5
part 2

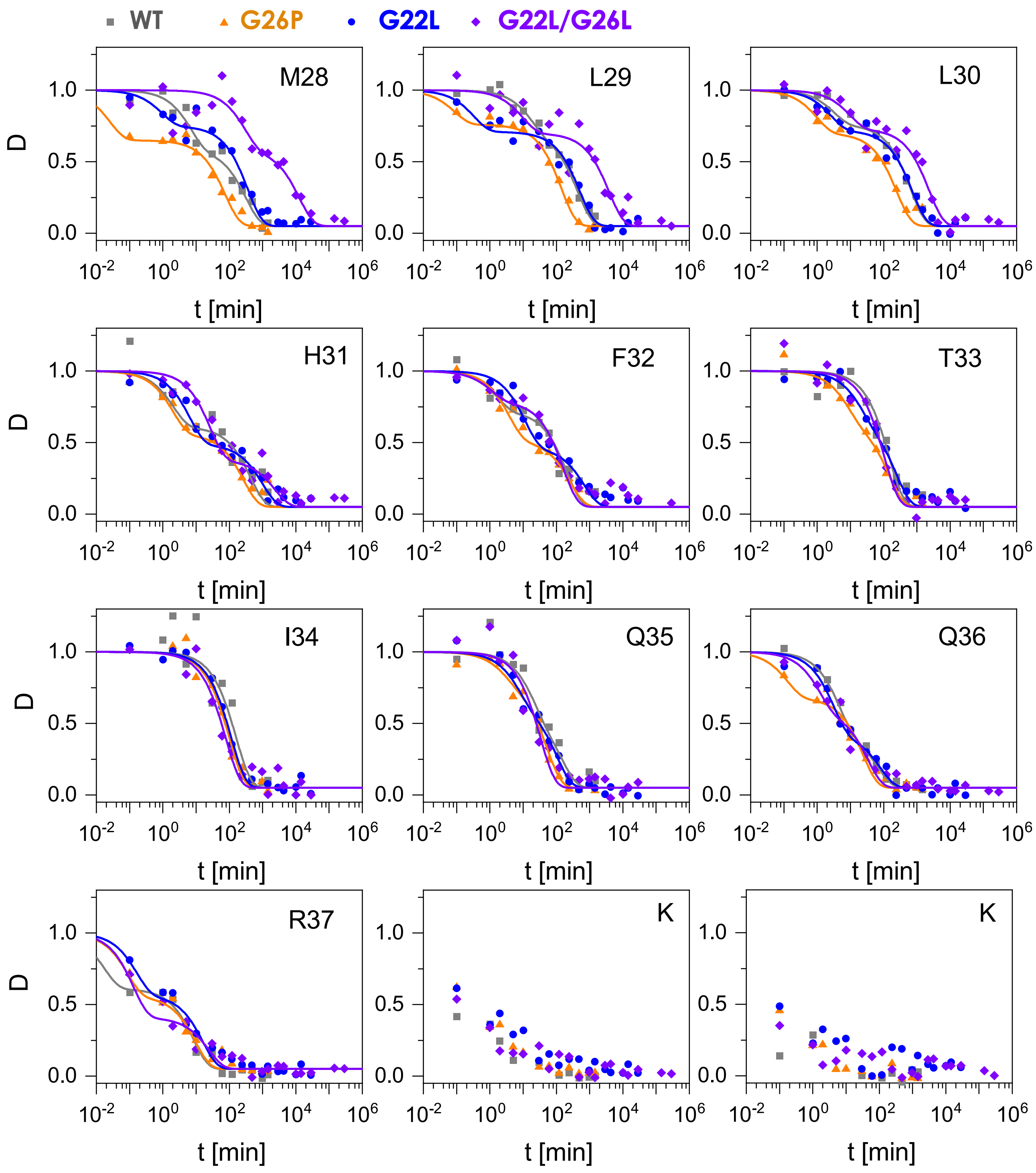

Supplementary Figure 5. Residue-specific amide DHX kinetics. The calculated deuterium contents D (mean values, n = 3) of the respective amides are plotted against the exchange period t. The kinetics were used to calculate the respective k_{exp} values (Fig. 4) after data fitting with monoexponential or biexponential decay functions (see Methods). Fitting was only performed for those kinetics that were deemed complete enough for calculating k_{exp}. Sequence positions and mutations are given in the insets.

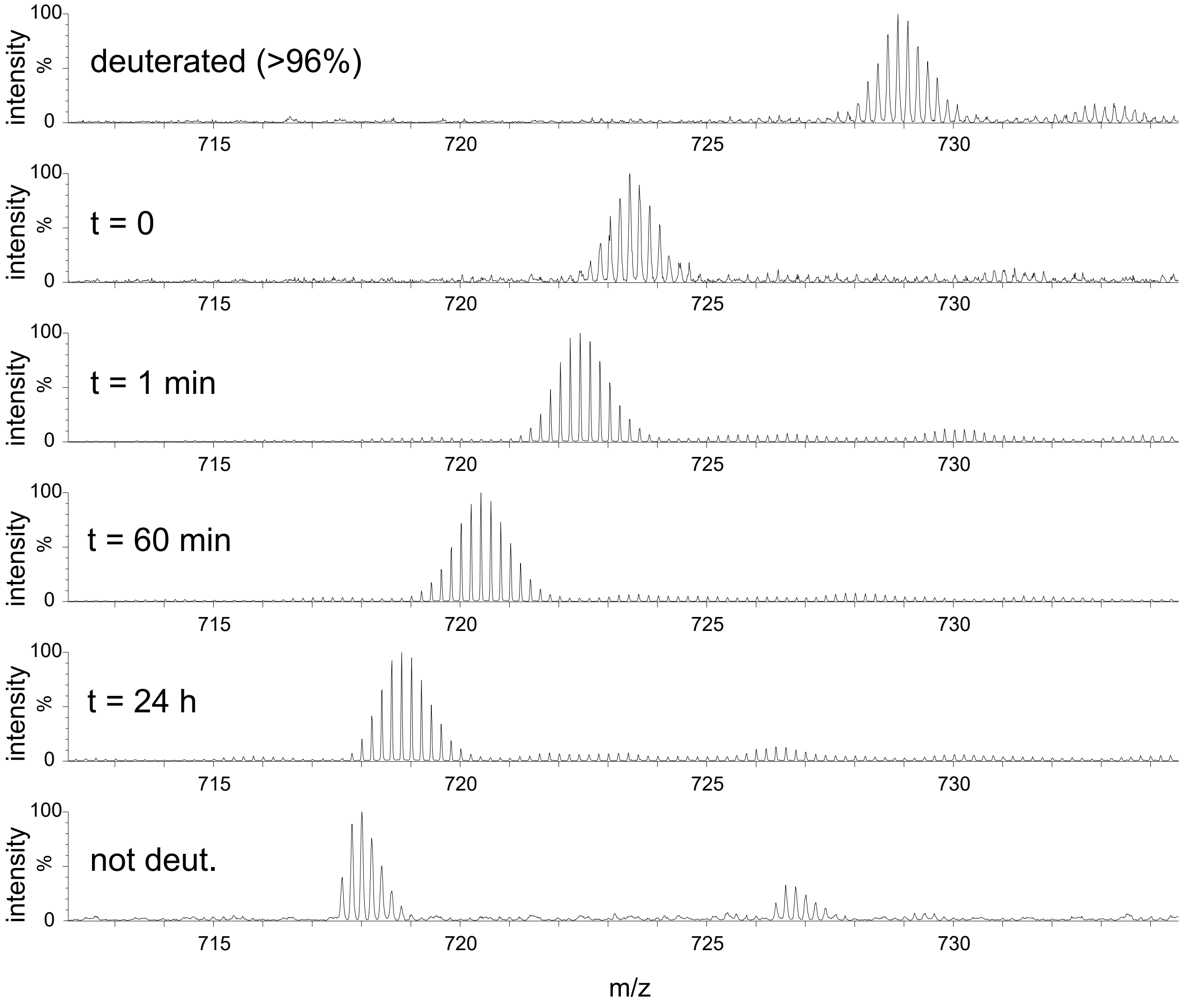

Supplementary Figure 6. Representative mass spectra of the 5+ charged GnTV WT peptide ion from different time points of a DHX experiment. Note the isotope pattern that gradually shifts with incubation time indicates a preferential EX2 mode of DHX.

Supplementary Figure 7

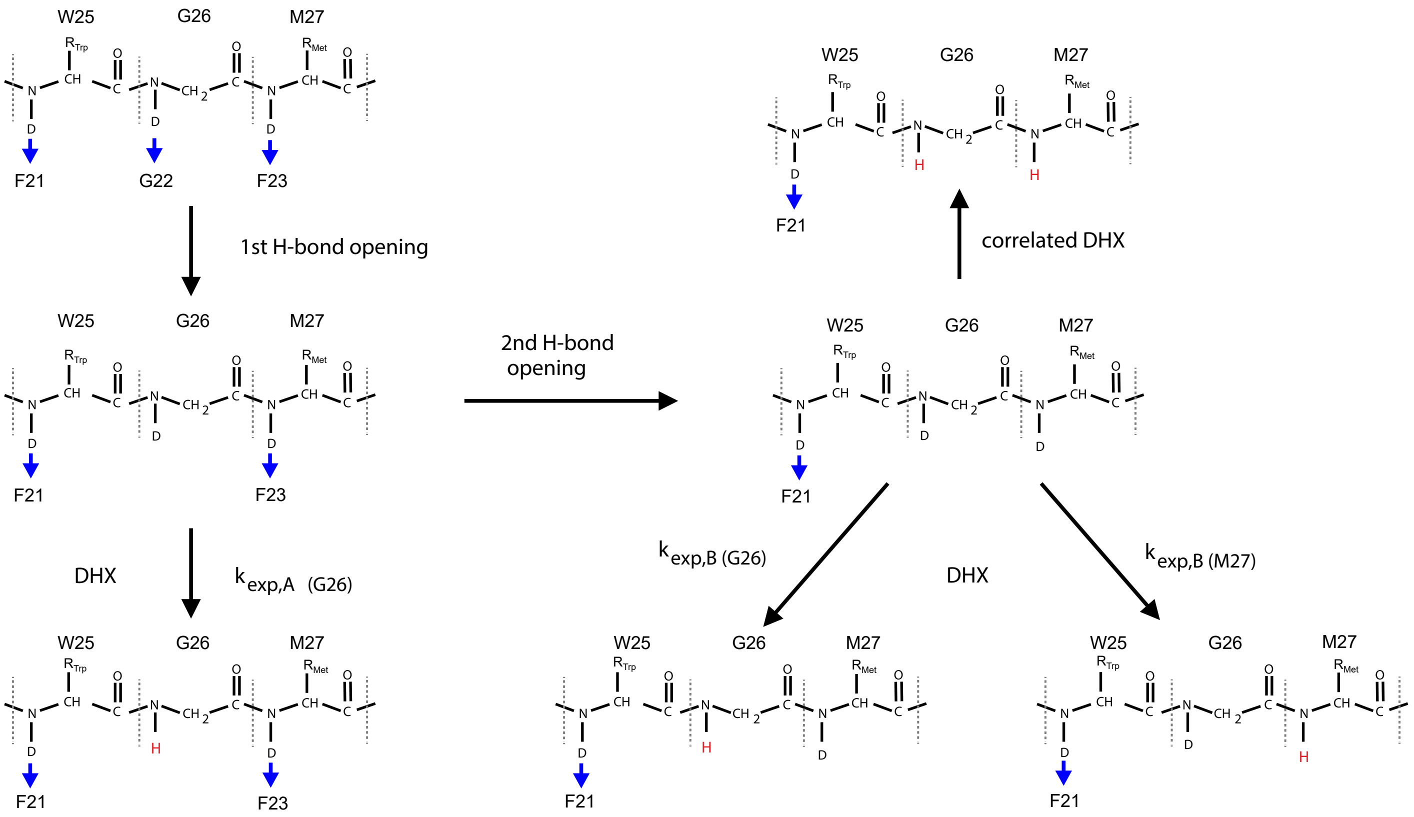

Supplementary Figure 7. The scheme illustrates how the loss of an (i,i-4) amide H bond (blue arrows) at G26 might allow fast DHX at G26. Alternatively, H-bond loss at G26 might lead to loss of a neighboring amide H-bond, e.g. at M27, followed by slower DHX at either M27 or G26. An alternative pathway (correlated DHX) may simultaneously exchange the amide Ds of G27 and M27 (see: supplementary discussion).

Supplementary Discussion

To explain the origin of biexponential DHX, we assume that exchange at a given amide within a population of TMDs can follow one of two different kinetic regimes. The fast regime yields a high $k_{exp,A}$ while the slow regime leads to a low $k_{exp,B}$ value. We propose that $k_{exp,A}$ and $k_{exp,B}$ values describe DHX at a given open amide ('single opening') or at simultaneously open neighboring amide pairs ('double opening'), respectively. Even in the latter case, i.e., after simultaneous opening of two H-bonds, less than one exchange event can occur per opening on average, given that open/close transitions of an H-bond are much more frequent than the chemical amide exchange reaction of an unfolded peptide in the EX2 mode (Zheng et al. 2019). That the EX2 regime characterizes the bulk of DHX events of the GnTV TM domain under our conditions is shown by isotope patterns whose masses are shifting with time in a gradual fashion, rather than forming a bimodal shape (Suppl. Fig. 6).

For example, H-bond opening at G26 may result in local DHX with a fast $k_{exp,A}$ and/or facilitate a second H-bond opening at W25 or M27. That second opening may enable DHX at W25 or M27, albeit with a slow $k_{exp,B}$, as a double opening is likely a rare event (Suppl. Fig. 7). Exchange from the same doubly open state may also occur at G26, provided that a previous opening at G26 had not resulted in DHX. Accordingly, we propose that $k_{exp,A}$ represents frequent single openings while $k_{exp,B}$ may describe correlated double openings occurring at lower frequencies.

This proposition is based on the expectation that isolated openings are more frequent than reactions leading to two simultaneously open amides. We cannot rule out an inverse mechanism, however, where fast DHX occurs at two simultaneously open amides and slow DHX at isolated openings. This alternative interpretation would hold true if the decrease in the closing rate from a doubly open state would overcompensate a decrease in the opening rate leading to it. In this case, the aggregate lifetime of a doubly open state might exceed the aggregate lifetime of isolated openings.

In another hypothetical model, the postulated rare double openings are stable enough for correlated DHX to occur at neighboring amides at frequencies that are too low to visibly distort the pattern of gradual exchange of the parent ion (Suppl. Fig. 6). As a result, exchanges of a single D at a highly flexible region of a helix, such as a hinge or a frayed terminus, may be 'contaminated' with a low fraction of double exchanges. Since the contaminating double exchanges simulate two consecutive non-correlated DHX events when fitting the resulting asymmetric isotopic envelopes of the peptides after ETD fragmentation, they would mimic accelerated exchange. In this model, accelerated exchange is mimicked only for the initial periods of the exchange reaction, however, where neighboring deuteron pairs can give rise to correlated DHX. At later time points, correlated DHX/HDX is not expected to yield kinetics that can distinguished from non-correlated DHX. This alternative model is also suited to explain fast initial kinetics followed by slower kinetics at flexible helix regions. Asymmetrical isotope patterns have previously been observed in situations of mixed EX1/EX2 exchange (Abzalimov and Kaltashov, 2006).

Importantly, both models are equally consistent with our conclusions on the relevance of local helix flexibility for cleavage by γ-secretase.

Supplementary Figure 8

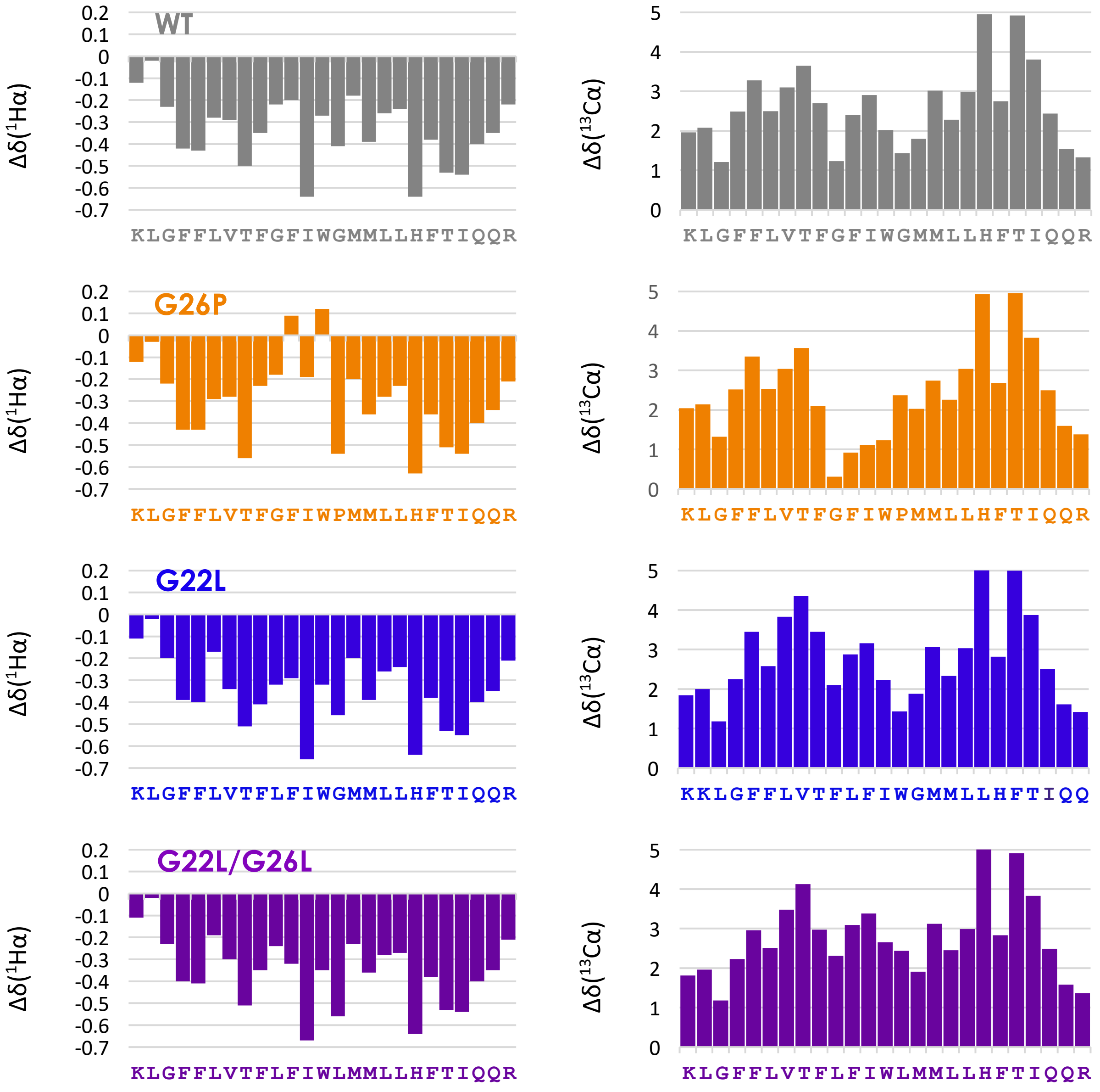

Supplementary Figure 8. ^{1}Ha and ^{13}Ca secodary chemical shifts.

Supplementary Figure 9

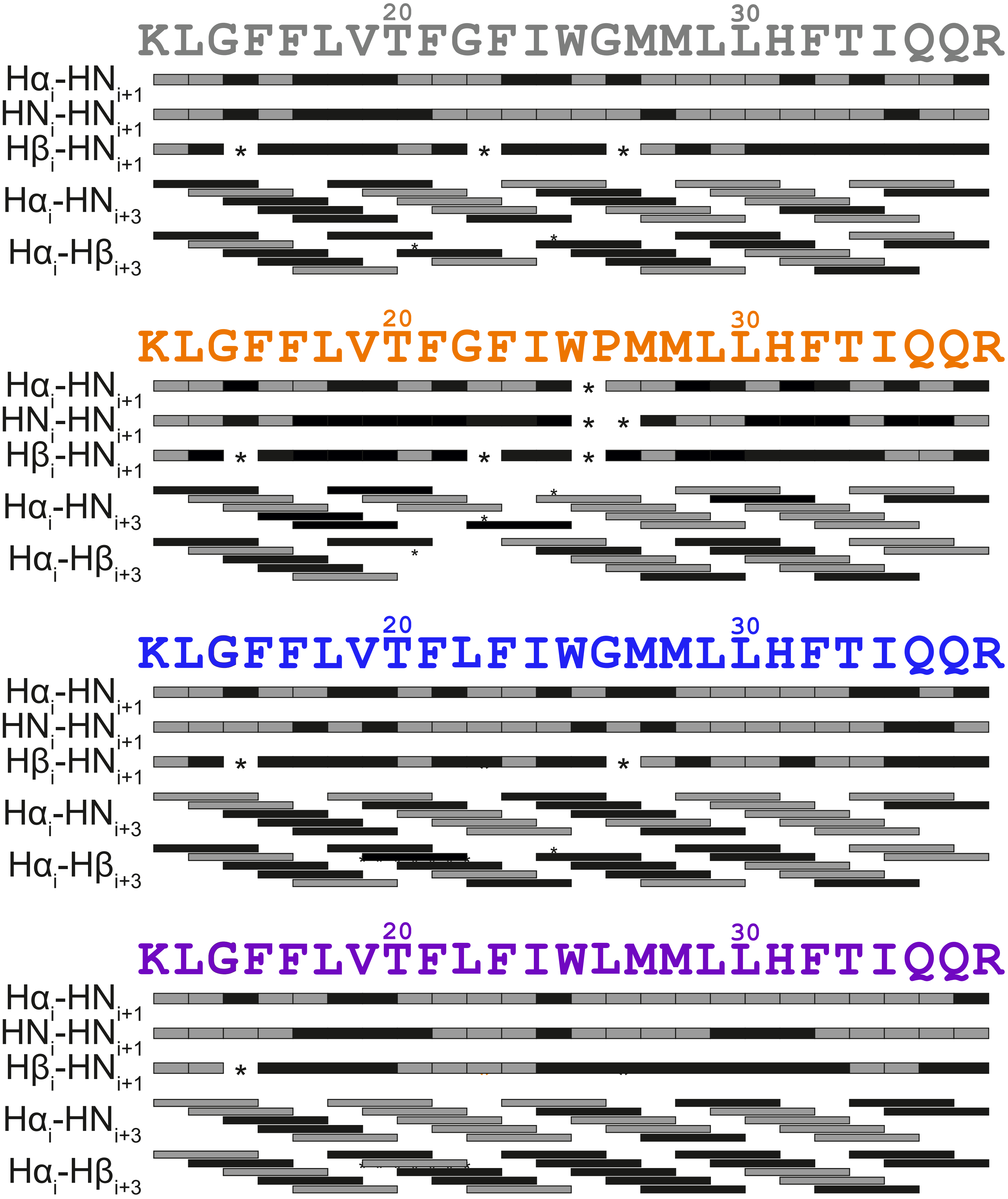

Supplementary Figure 9. NOE contacts between the respective 1H. Black bars indicate unambiguous NOE, grey bars overlapping signals and asterisks lack of the respective Hβ. GnTV WT amino acid sequence in grey, GnTV G26P in orange, GnTV G22L in blue and GnTV G22L/G26L in purple.

Supplementary Figure 10

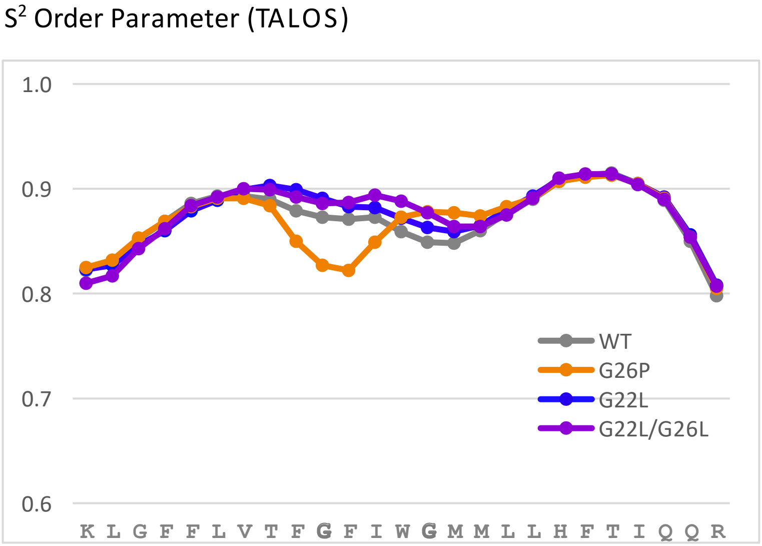

Supplementary Figure 10. S^2 order parameters determined with TALOS+ based on chemical shift data.

Supplementary Table 1 Sequences of synthetic GnTV peptides[1]

Peptide	Sequence	Average mass [Da]
**WT**	Ac-KKKLGFFLVTFGFIWGMMLLHFTIQQRKK-NH_2	3585.48
**G26P**	Ac-KKKLGFFLVTFGFIW**P**MMLLHFTIQQRKK-NH_2	3625.54
**G22L**	Ac-KKKLGFFLVTF**L**FIWGMMLLHFTIQQRKK-NH_2	3641.59
**G22L/ G26L**	Ac-KKKLGFFLVTF**L**FIW**L**MMLLHFTIQQRKK-NH_2	3697.69

[1] The hydrophobic sequences are flanked by lysine triplets for better solubility and gas phase fragmentation, N-termini are acetylated and C-termini are amidated.

Supplementary Table 2 Structure statistics of GnTV WT and mutant TM domains

	**WT**	**G22L**	**G22L/G26L**	**G26P**
**Total restraints used**				
unambiguous NOE restraints	277	261	267	286
Intraresidue	145	113	141	126
Sequential (|i-j|=1)	64	62	59	86
Medium range (1 < |i-j|< 4)	57	74	55	53
Long range (|i-j|≥ 4)	11	12	12	21
Ambiguous NOE restraints	39	39	49	55
**Statistics for structure calculations**				
RMSD of bonds (A)	0.001+/- 0.00005	0.001 +/- 0.00007	0.001 +/- 0.00006	0.001 +/- 0.0004
RMSD of bond angles (°)	0.292 +/- 0.004	0.308 +/- 0.007	0.308 +/- 0.005	0.316 +/- 0.006
RMSD of improper torsions (°)	0.138 +/- 0.009	0.153 +/- 0.017	0.145 +/- 0.015	0.144 +/- 0.01
**Final Energies (kcal mol^{-1})**				
E_{total}	-978.5 +/- 41.3	-991.0 +/- 37.7	-1004 +/- 35	-969.1 +/- 37.7
E_{bonds}	0.550 +/- 0.060	0.655 +/- 0.082	0.570 +/- 0.068	0.817 +/- 0.053
E_{angles}	12.55 +/- 0.37	14.36 +/- 0.73	14.65 +/- 0.44	15.02 +/- 0.56
$E_{impropers}$	0.855 +/- 0.115	1.06 +/- 0.24	0.965 +/- 0.201	0.936 +/- 0.169
E_{dihed}	128.7 +/- 3.00	132.4 +/- 2.6	137.9 +/- 3.2	127.4 +/- 2.2
E_{vdW}	-244.7 +/- 2.9	-253.8 +/- 4.02	-262.33 +/- 3.22	-254.7 +/- 3.4
E_{NOE}	-876.5 +/- 41.3	-885.7 +/- 37.5	-895.9 +/- 34.4	-1858.6 +/- 36.1
**Coordinate precision (A)**				
RMSD of backbone (N,CA,C,O) of all residues	1.97 +/- 0.73	1.57 +/- 0.64	2.39 +/- 0.90	2.81 +/- 1.66
RMSD of all heavy atoms of all residues	2.76 +/- 0.85	2.47 +/- 0.73	2.84 +/- 0.83	3.25 +/- 1.60

All values refer to the ensemble of 50 structures with the lowest energy from 400 calculated structures.

Figure 2 B raw

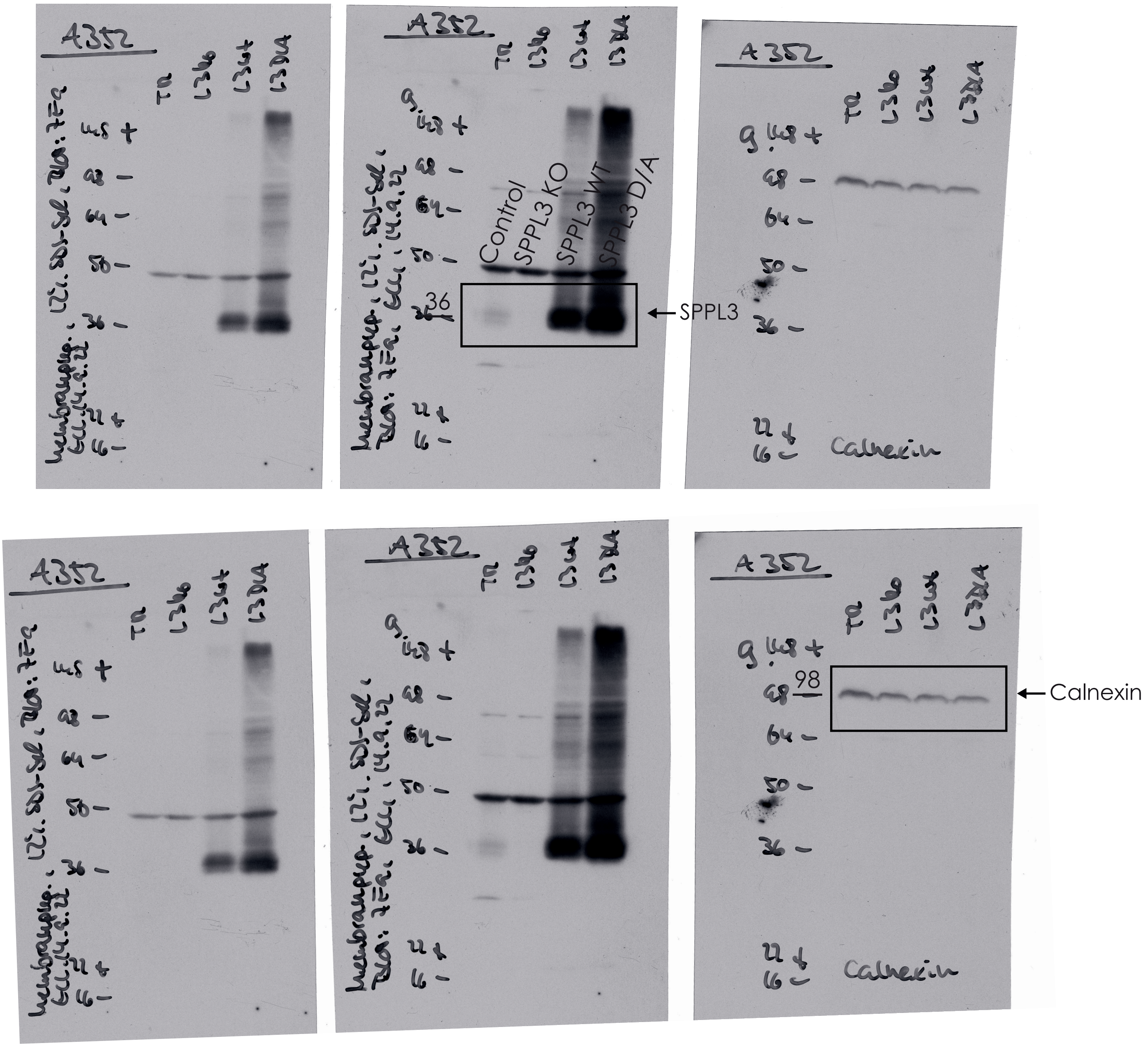

Supplementary Figure 1 Part 1

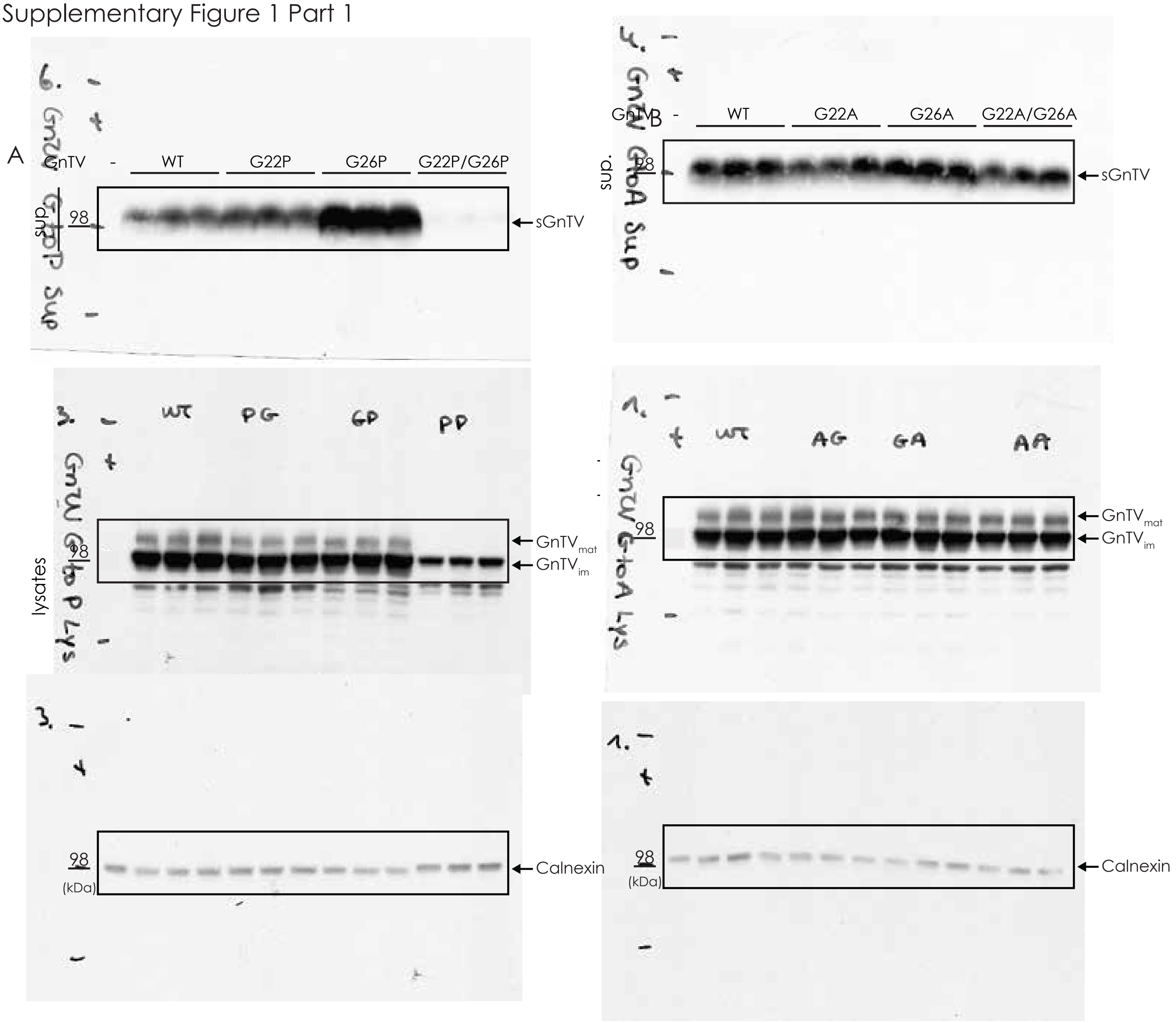

Supplementary Figure 1 Raw

Supplementary Figure 1 Part 2

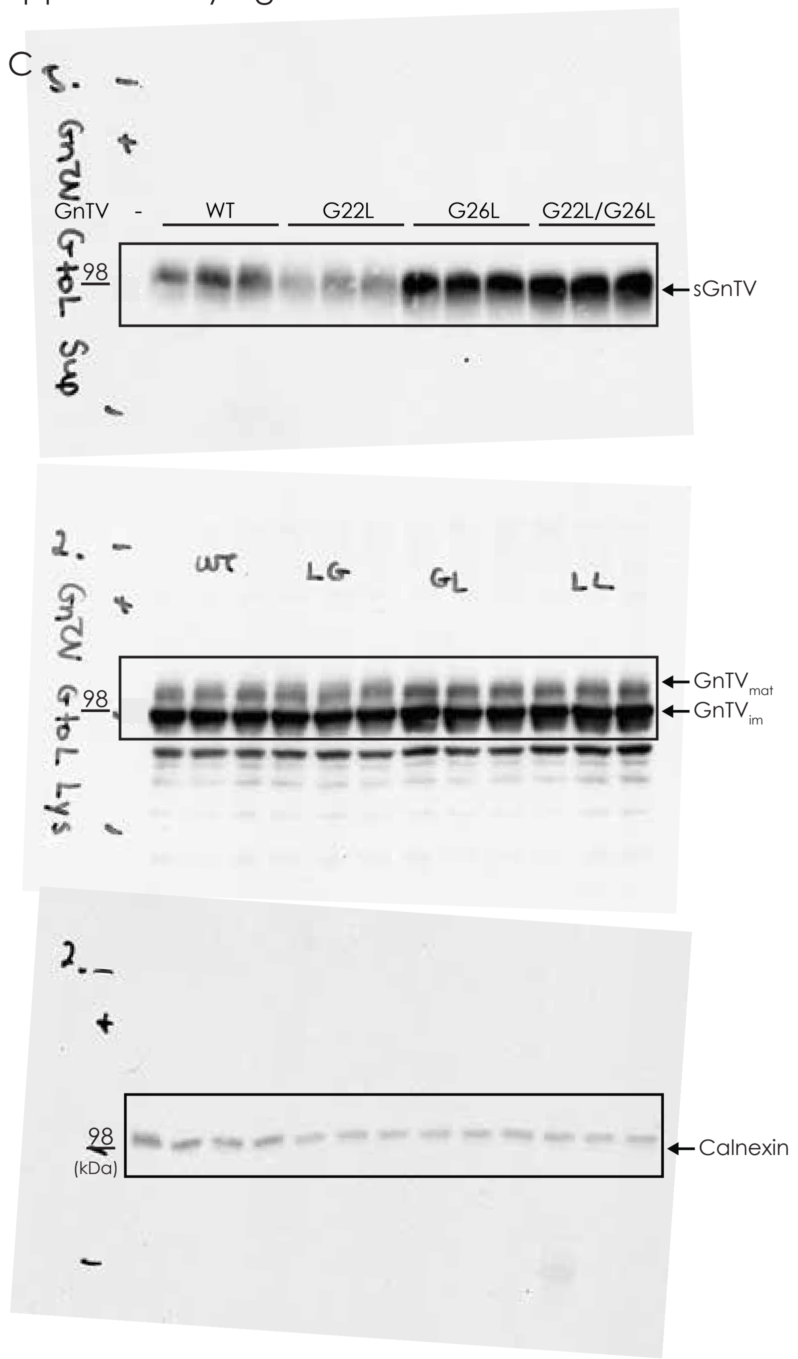

Supplementary Figure 1 Raw

Supplementary Figure 2

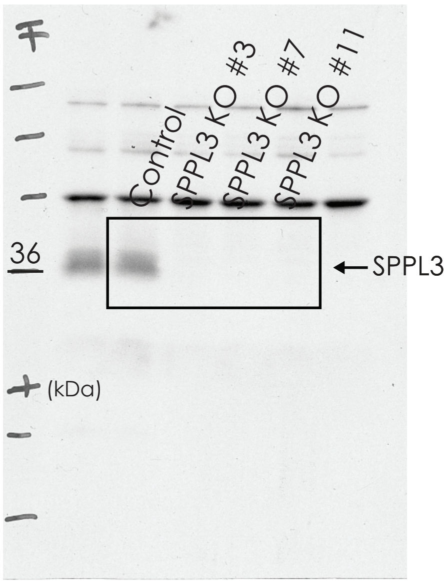

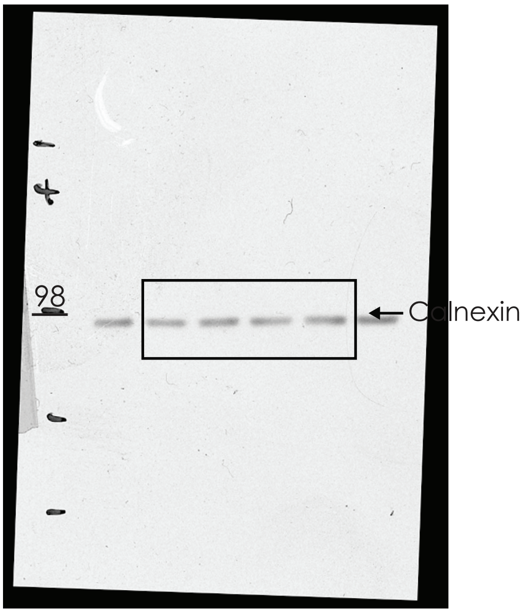

Supplementary Figure 2 Raw
